# The ribonuclease DIS3 promotes *let-7* miRNA maturation by degrading the pluripotency factor *LIN28B* mRNA

**DOI:** 10.1093/nar/gkv387

**Published:** 2015-04-29

**Authors:** Simona Segalla, Silvia Pivetti, Katia Todoerti, Malgorzata Agata Chudzik, Erica Claudia Giuliani, Federico Lazzaro, Viviana Volta, Dejan Lazarevic, Giovanna Musco, Marco Muzi-Falconi, Antonino Neri, Stefano Biffo, Giovanni Tonon

**Affiliations:** 1 Functional Genomics of Cancer Unit, Division of Experimental Oncology, Istituto di Ricovero e Cura a Carattere Scientifico (IRCCS) San Raffaele Scientific Institute, 20133 Milan, Italy; 2 Laboratory of Pre-Clinical and Translational Research, IRCCS-CROB, Referral Cancer Center of Basilicata, 85028 Rionero in Vulture, Italy; 3 Dipartimento di Bioscienze, Università degli Studi di Milano, 20132 Milan, Italy; 4 Molecular Histology and Cell Growth Laboratory, Istituto di Ricovero e Cura a Carattere Scientifico (IRCCS), San Raffaele Science Institute, 20132 Milan, Italy; 5 Center for Translational Genomics and Bioinformatics, Istituto di Ricovero e Cura a Carattere Scientifico (IRCCS), San Raffaele Scientific Institute, 20132 Milan, Italy; 6 Dulbecco Telethon Institute, S. Raffaele Hospital, 20132 Milan, Italy; 7 Department of Clinical Sciences and Community Health, University of Milan, Hematology1 CTMO, Foundation IRCCS Ca’ Granda Ospedale Maggiore Policlinico, 20122 Milan, Italy; 8 Dipartimento di Scienze e Innovazione Tecnologica, University of Piemonte Orientale, 15100 Alessandria, Italy; 9 Università Vita-Salute San Raffaele, Milan, 20132, Italy

## Abstract

Multiple myeloma, the second most frequent hematologic tumor after lymphomas, is an incurable cancer. Recent sequencing efforts have identified the ribonuclease DIS3 as one of the most frequently mutated genes in this disease. DIS3 represents the catalytic subunit of the exosome, a macromolecular complex central to the processing, maturation and surveillance of various RNAs. miRNAs are an evolutionarily conserved class of small noncoding RNAs, regulating gene expression at post-transcriptional level. Ribonucleases, including Drosha, Dicer and XRN2, are involved in the processing and stability of miRNAs. However, the role of DIS3 on the regulation of miRNAs remains largely unknown. Here we found that DIS3 regulates the levels of the tumor suppressor *let-7* miRNAs without affecting other miRNA families. DIS3 facilitates the maturation of *let-7* miRNAs by reducing in the cytoplasm the RNA stability of the pluripotency factor LIN28B, a inhibitor of *let-7* processing. DIS3 inactivation, through the increase of LIN28B and the reduction of mature *let-7*, enhances the translation of *let-7* targets such as MYC and RAS leading to enhanced tumorigenesis. Our study establishes that the ribonuclease DIS3, targeting LIN28B, sustains the maturation of *let-7* miRNAs and suggests the increased translation of critical oncogenes as one of the biological outcomes of DIS3 inactivation.

## INTRODUCTION

DIS3 encodes a ribonuclease endowed with two different RNase activities: a 3′-5′ exonucleolytic activity via the RNase II/R (RNB) domain and an endonucleolytic activity via the PilT N-terminal (PIN) domain (1). DIS3 is the catalytic subunit of exosome, a multiprotein complex present in both the nucleus and the cytoplasm, which plays a crucial role in processing, quality control and turnover of a large number of cellular RNAs (2,3). The core of the eukaryotic exosome is composed of nine subunits that form a cylindric, barrel-like structure with a prominent central channel (2,4,5). Six of the subunits share overall structural similarity with the bacterial phosphorolytic nuclease RNase PH and assemble into a hexameric ring. The other three subunits are positioned on one side of the ring and are characterized by S1/KH domains often found in RNA-binding proteins. The eukaryotic core-exosome displays RNA-binding properties but lacks enzymatic activity, which is provided by DIS3. DIS3 binds at the distal end of the barrel relative to the S1 and KH ring and provides catalytic activity to the entire ten-subunit complex (4,5).

Recent next-generation sequencing efforts have identified somatic mutations in *DIS3* in the hematological cancers multiple myeloma (MM) and acute myeloid leukemia. In MM, in particular, up to 18% of MM patients present mutations affecting this gene. *DIS3* mutations are located for the most part within the major ribonuclease domains of the protein, suggesting the mutations impair the enzymatic activity of DIS3 (6,7). Indeed, mutant strains in yeast harboring nucleotide substitutions corresponding to the mutations detected in human patients show growth inhibition and changes in nuclear RNA metabolism consistent with the dysfunction of the exosome (8). In addition *in vitro* assays have shown that yeast DIS3 mutants have a reduced ability to exonucleolytically digest RNA substrates (8). The reduced activity of yeast DIS3 mutants alongside genomic data, that show how tumor cells usually retain only the mutated copy of *DIS3*, suggest a role for DIS3 as a tumor suppressor in multiple myeloma (7). However, how loss-of-function DIS3 mutations are tumorigenic and how they contribute to multiple myeloma pathogenesis remains largely unknown.

MicroRNAs (miRNAs) are an evolutionarily conserved class of small (18–22 nucleotides) noncoding, single stranded RNAs that regulate gene expression at post-transcriptional level by binding the 3′-untranslated (3′ UTR) region of mRNAs and inducing translational inhibition and/or degradation of their targets (9). miRNAs are transcribed by RNA polymerase II that produces long primary miRNA transcripts (pri-miRNAs) that subsequently undergo post-transcriptional modifications. In the nucleus, the RNaseIII enzyme Drosha crops the pri-miRNA into a 70 nucleotide (nt) hairpin-structured precursor (pre-miRNA). Pre-miRNA is then exported to the cytoplasm where it is cleaved by another RNaseIII enzyme, Dicer, that removes the ‘terminal loop region’, finally yielding the mature 22 nt miRNA (10). The tight control of miRNA biogenesis, both at the transcriptional and post-transcriptional level, is critical for the proper functioning of a variety of central biological processes including development, differentiation (11) and hematopoiesis (10). Deregulation of miRNA expression has been associated with multiple diseases including cancer (12,13) where a global reduction of miRNAs has been often observed as a general trait (14–16). Notably, this repression is not due to a reduction in the transcription of primary miRNA species, suggesting a critical role of miRNA processing in tumorigenesis (17).

Among miRNAs, *let-7* represents an important family. It includes 12 members residing in various locations throughout the genome that are frequently deleted in human cancers. The *let-7* miRNA family members act as tumor suppressors. They negatively regulate the translation of oncogenes and cell cycle regulators including RAS, MYC and HMGA2 (11,18,19). *let-7* miRNAs present decreased expression in several cancer types, and low *let-7* levels correlate with poor prognosis (20,21). Conversely, overexpression of *let-7* blocks cellular proliferation, inhibits cell growth and impairs tumor development in a xenograft model of non-small cell lung cancer (22).

The pluripotency factor LIN28 reduces *let-7* expression, blocking its maturation (22,23). Reducing *let-7*, LIN28 maintains the undifferentiated and proliferative state of stem cells (24). LIN28 is highly expressed during embryogenesis and then silenced in adult somatic cells. There are two paralogues of LIN28 (LIN28A and LIN28B) structurally similar but distinguishable in expression patterns, subcellular localization and mechanism leading to *let-7* inhibition (25–27). LIN28A acts in the cytoplasm, recruiting a TUTase, ZCCHC11 to *let-7* precursors and hampering their processing by Dicer (28). On the contrary, LIN28B sequesters immature *let-7* transcripts in the nucleus, inhibiting their further processing by Microprocessor (27). LIN28A and LIN28B exert prominent roles in stem cell biology, tissue development, and also in tumorigenesis (29). LIN28A and B untimely and inappropriate re-expression has been reported in several cancer type (29). They are often overexpressed in a mutually exclusive manner, and behave as oncogenes largely through their repression of *let-7* miRNAs (30).

In this study, we aimed to explore and define the role of DIS3 on miRNA biogenesis. Our findings point to a crucial role for the ribonuclease DIS3 in promoting the maturation of the *let-7* miRNA tumor suppressor family, through a DIS3-mediated control of the pluripotency factor LIN28B.

## MATERIALS AND METHODS

### Cell culture and transfections

U2OS, HEK-293T and NIH3T3 cells were cultured in Dulbecco's modified Eagle's medium (DMEM) supplemented with 10% fetal bovine serum (FBS) and 1% of Penicillin and Streptomycin antibiotics. RPMI-8226 and KMS12-BM cells were cultured in RPMI medium with 10% FBS and 1% of Penicillin and Streptomycin. To silence *SKI2* and *MTR4* we used Dharmacon SMART pool siRNAs (Dharmacon, Lafayette, CO, USA) specific for *SKI2* (cat. no. L-013435-01-0010), for *MTR4* (cat. no. L-031902-02-0010) or a SMART pool of scrambled siRNAs (cat. no. D-001810-01-20) as control. To overexpress miRNA *let-7a* U2OS cells were transfected with miRNA mimic of *let-7a* (Ambion, Naugatuck, CT, USA; cat.no. 4464066) or a miRNA mimic negative control (cat. no. 4464058). siRNAs and miRNA mimics were transfected in U2OS cells by lipofectamine RNAiMAx (Invitrogen, Carlsbad, CA, USA) at a final concentration respectively of 30 and 33 nM according to the manufacturer's instructions.

### Lentiviral vectors

To knockdown DIS3 expression, pLKO.1 lentiviral vectors carrying short hairpin RNAs targeting human or murine *DIS3* were used in infection experiments. Non-targeting, scrambled shRNA (ctrl shRNA) was used as negative control. For further details see supplementary methods.

### Western blotting

The protocol, the antibodies and working dilutions are described in details in the supplementary methods.

### RNA extraction, fractionation and qRT-PCR

Total RNA was extracted from cells using TRIzol^®^ RNA Isolation Reagent (Invitrogen, Carlsbad, CA, USA) and transcripts were quantified by q-RT-PCR using comparative threshold cycle method. Further details are provided in the supplementary methods.

### mRNA and miRNA expression profiling

Total RNA from RPMI-8226 and KMS12-BM cell lines knocked-down with a scrambled shRNA (control sh) or *DIS3* shRNA (DIS3 sh) was collected 72 h after infection and extracted with TRIzol^®^ RNA Isolation Reagent. Quality assessment was performed using a Bioanalyzer Agilent 2100 (Agilent, Santa Clara, CA, USA). Total RNA samples were processed using the FlashTag labeling kit and then hybridized to the GeneChip^®^ miRNA arrays v1.0 (Affymetrix Inc., Santa Clara, CA, USA), according to the Affymetrix recommended protocol. Expression values for 847 human miRNAs were extracted from CEL ﬁles using Affymetrix miRNA QC tool software (RMA normalized and log_2_-transformed (31)). Data were analyzed using the software dCHIP (http://biosun1.harvard.edu/complab/dchip). The microarray data have been deposited in the Gene Expression Omnibus (GEO) under accession number GSE55246 (available at http://www.ncbi.nlm.nih.gov/geo/query/acc.cgi?token=orydwgscvrmpbaz&acc=GSE55246). Gene set enrichment analysis (GSEA) was performed as previously described (GSEA v2.0 at http://www.broad.mit.edu/gsea, (32)) using gene set as permutation type and 1000 permutations and signal to noise as metric for ranking genes. miRNA families were derived from miRBase (http://www.mirbase.org). In line with GSEA default settings, only miRNA families including at least five members were included in the analysis.

### RNA stability assay

U2OS infected with a scrambled shRNA or a shRNA specific for *DIS3* were treated with 5,6-dichloro-ribofuranosylbenzimidazole (DRB, 100 μg/ml, Sigma, St. Louis, MO, USA), an RNA polymerase II inhibitor. Cells were collected at 0, 2, 3, 4 h after treatment and RNA extracted using RNeasy microKIT (Qiagen, Hilden, Germany). Reverse transcription was performed with random primers and mRNA levels were measured by qRT-PCR. Primers sequences are provided in Supplemental Table S3.

### Cycloheximide treatment

U2OS infected with a scrambled shRNA, as control, or with a shRNA specific for DIS3 were puromycin selected and, 72 h after infection, were treated with 100 μg/ml of cycloheximide for 0.5, 1, 2, 3 h. Cell lysates were blotted for MYC and LaminB.

### Focus formation assay

Focus formation assay of NIH3T3 cells infected with scrambled shRNA (as control) or with shRNA specific for *DIS3* were plated at 60 000 cells for six-well, grown for 21 days, fixed and stained with crystal violet.

## RESULTS

### Loss of DIS3 specifically decreases *let-7* miRNA levels, without affecting other miRNA families

**Figure 1.**
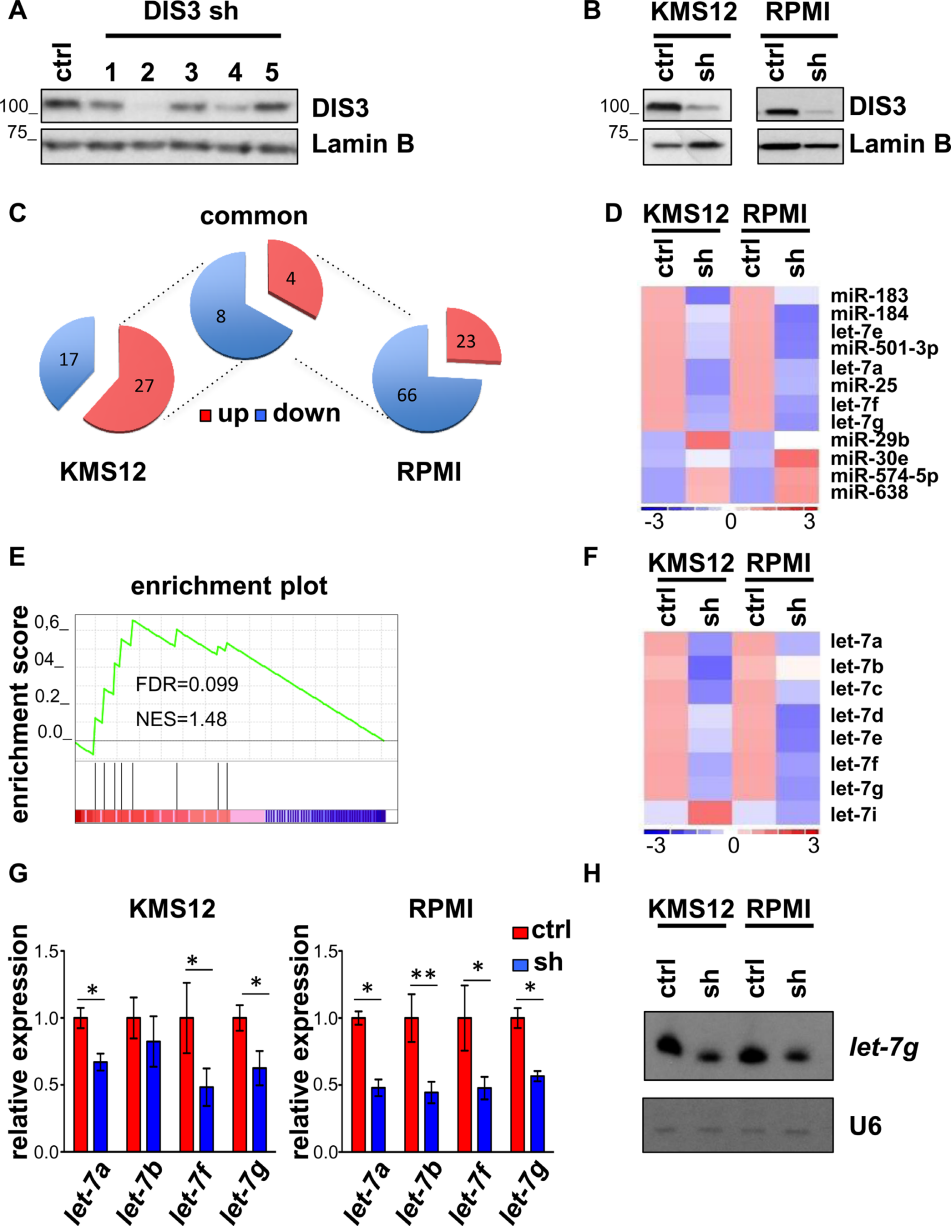
*DIS3* knockdown in MM cells selectively affects *let-7* miRNA family. (**A**) Western blot to test the efficiency of five different shRNAs to silence *DIS3* in HEK-293T cell line. (**B**) Western blot analysis of DIS3 expression in MM cell lines, KMS12-BM (KMS12) AND RPMI-8226 (RPMI), infected with a scrambled shRNA (ctrl) or *DIS3* shRNA4 (shRNA). Lamin B was used as loading control. (**C**) Pie charts summarizing the number of miRNAs deregulated >1.5-fold in KMS12 and RPMI, and in both cell lines after *DIS3* knockdown. (**D**) Heat map showing the expression levels of miRNA varying >1.5-fold in both KMS12 and RPMI upon *DIS3* silencing. The color scale bar in the heat map represents the relative miRNA expression with red representing up-regulation and blue representing down-regulation. (**E**) GSEA ES plot showing the enrichment of *let-7* miRNA family gene set after *DIS3* silencing. (**F**) Heat map of *let-7* miRNA family levels in KMS12 and RPMI cell lines after infection with a scrambled shRNA (ctrl) or *DIS3*-specific shRNA4 (sh). (**G**) Expression of the *let-7* miRNA members (*a, b, f, g*) assayed by qRT-PCR in KMS12 and RPMI cell lines infected with scrambled (ctrl) or *DIS3*-specific shRNA4 (sh), 72 h after infection. Results are normalized over *RNU6B*. Bars represent SDs (*n* = 2 indipendent experiments). *P < 0.05; **P < 0.005 using two-tailed Student's *t* test. (**H**) Northern blot analysis of endogenous *let-7g* in KMS12 and RPMI cell lines infected with scrambled (ctrl) or *DIS3*-specific shRNA4 (sh), 72 h after infection. *RNU6B* was used as loading control.

To investigate the potential role of DIS3 in miRNA regulation, we first explored miRNA changes resulting from *DIS3* inactivation. To this end, we tested five shRNAs targeting *DIS3* in HEK-293T cells and obtained a robust knockdown with two hairpins, shRNA2 and shRNA4 (Figure 1A). We then infected two different MM cell lines, KMS12-BM (KMS12) and RPMI-8226 (RPMI), with *DIS3* shRNA4 or a scrambled shRNA, as control (Figure 1B). The RNA obtained from these cells was then hybridized to miRNA arrays, where their differential expression was assessed upon *DIS3* down-regulation. Remarkably, only a small fraction of the miRNAs present on the platform was impacted by *DIS3* knockdown (Figure 1C). Also, to our surprise, the number of miRNAs up-regulated after *DIS3* inactivation was more than compensated by the miRNAs that were down-regulated, suggesting that DIS3 might not directly control miRNA levels. In all, four miRNAs were up-regulated and eight were down-regulated 1.5-fold or more in both cell lines. Intriguingly, among the miRNAs down-regulated, there were several members of the *let-7* family (Figure 1D). To obtain a more rigorous assessment of the miRNA families impacted by DIS3, a Gene Set Enrichment Analysis (GSEA) was performed (32). Remarkably, the only pathway that was significantly dysregulated as a result of DIS3 inactivation was indeed the *let-7* miRNA family (NES = 1.48, *P* < 0.05, FDR = 0.099, Figure 1E), with almost all the members of this family affected by *DIS3* knockdown. Intriguingly, however, *let-7* family members were down-regulated, and not up-regulated, as it would have been predicted given the ribonuclease activity of DIS3 (Figure 1F). To validate the array data, we chose four members of the *let-7* family (*let-7a, let-7b, let-7f, let-7g*) and we evaluated the effect of *DIS3* silencing on their expression by quantitative PCR (Figure 1G). Additionally, to further confirm the miRNA array data, we performed a northern blot to visualize the levels for one member of the *let-7* family (*let-7g*), in the same cells (Figure 1H). Consistent with the miRNA array results, probe for *let-7g* detected a reduction after DIS3 silencing. To rule out that *let-7* reduction was an off target effect of shRNA4, we evaluated *let-7* levels upon DIS3 knockdown with the second shRNA (shRNA2). ShRNA2-mediated silencing of *DIS3* reduced the levels of all *let-7* tested, as with shRNA4 (Supplementary Figure S1). All together, these data suggest that DIS3 does not pervasively regulate miRNAs, but rather impacts only on a very limited set of miRNAs, specifically affecting the miRNA family of tumor suppressor *let-7*.

**Figure 2.**
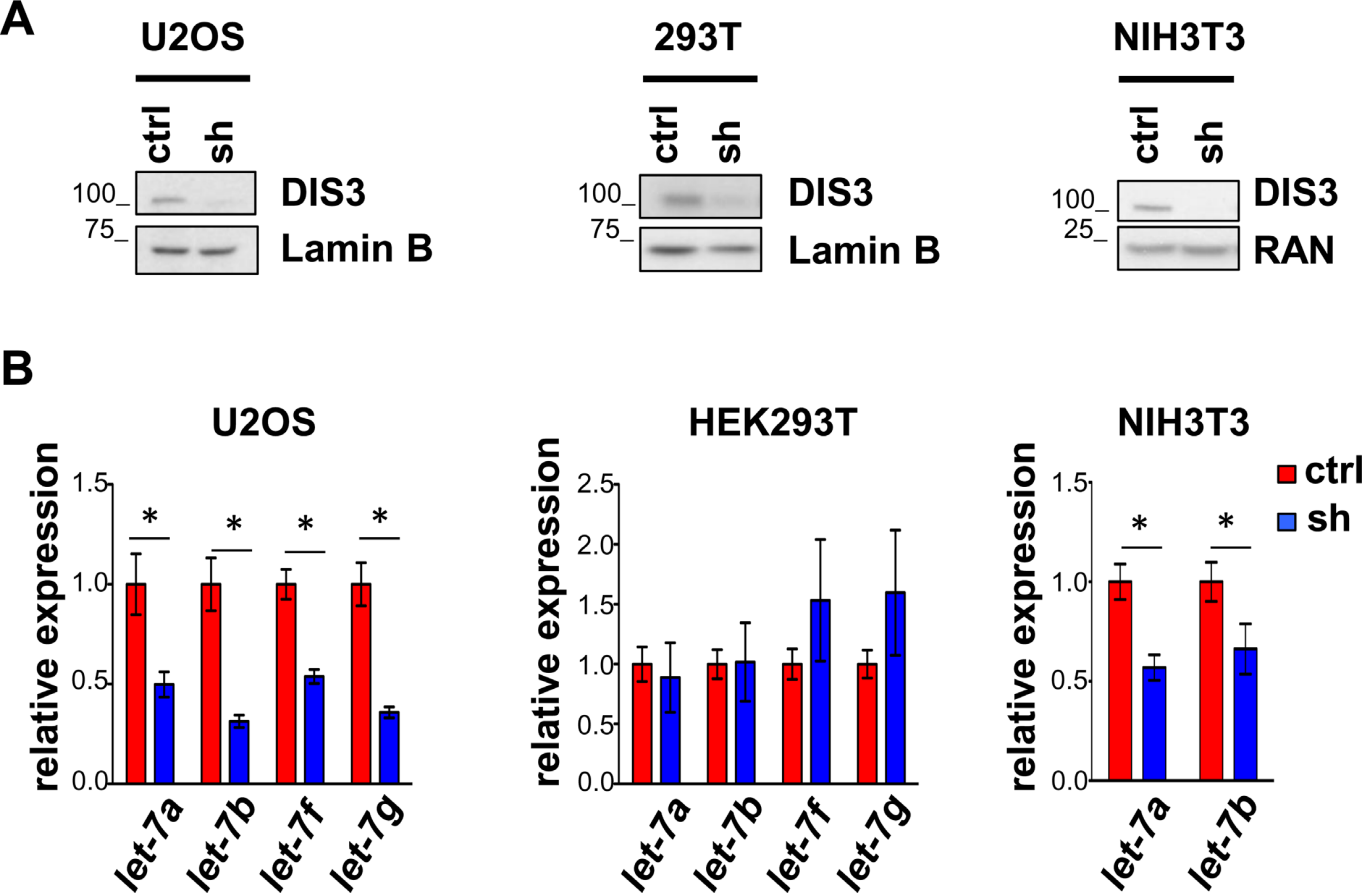
*let-7* levels after *DIS3* knockdown in human and mouse cell lines. (**A**) Western blot analysis of DIS3 expression in human U2OS and HEK293T cells, and in mouse NIH3T3 cells, 72 h after infection with scrambled (ctrl) and human or mouse *DIS3*-specific shRNAs (sh). Results are normalized over loading control Lamin B and RAN. (**B**) Expression of the *let-7* miRNA members was assayed by qRT-PCR. Results are normalized over *RNU6B* and *miR-16* respectively for human and mouse cells. Bars represent SDs (*n* = 2 indipendent experiments). *P < 0.05 using two-tailed Student's *t* test.

We next ascertained whether *let-7* modulation by DIS3 was a mechanism specific for MM or could be extended to unrelated cellular systems. We hence knocked down *DIS3* in human osteosarcoma U2OS cells, in human kidney embryonic HEK-293T cells and in mouse embryonic fibroblast NIH3T3 cells, using an shRNA targeting respectively the *DIS3* human (shRNA4) and *Dis3* mouse sequence (Figure 2A). qPCR demonstrated a strong reduction for *let-7a, let-7b, let-7f* and *let-7g* in U2OS and NIH3T3 cells, comparable to the one detected in MM cells (Figure 2B). Intriguingly, *DIS3* knockdown did not affect *let-7* levels in HEK-293T cells, suggesting a different molecular wiring relative to DIS3-mediated control on *let-7*, specific for this cell line.

*DIS3* down-regulation increases RAS and MYC protein levels through *let-7*.

**Figure 3.**
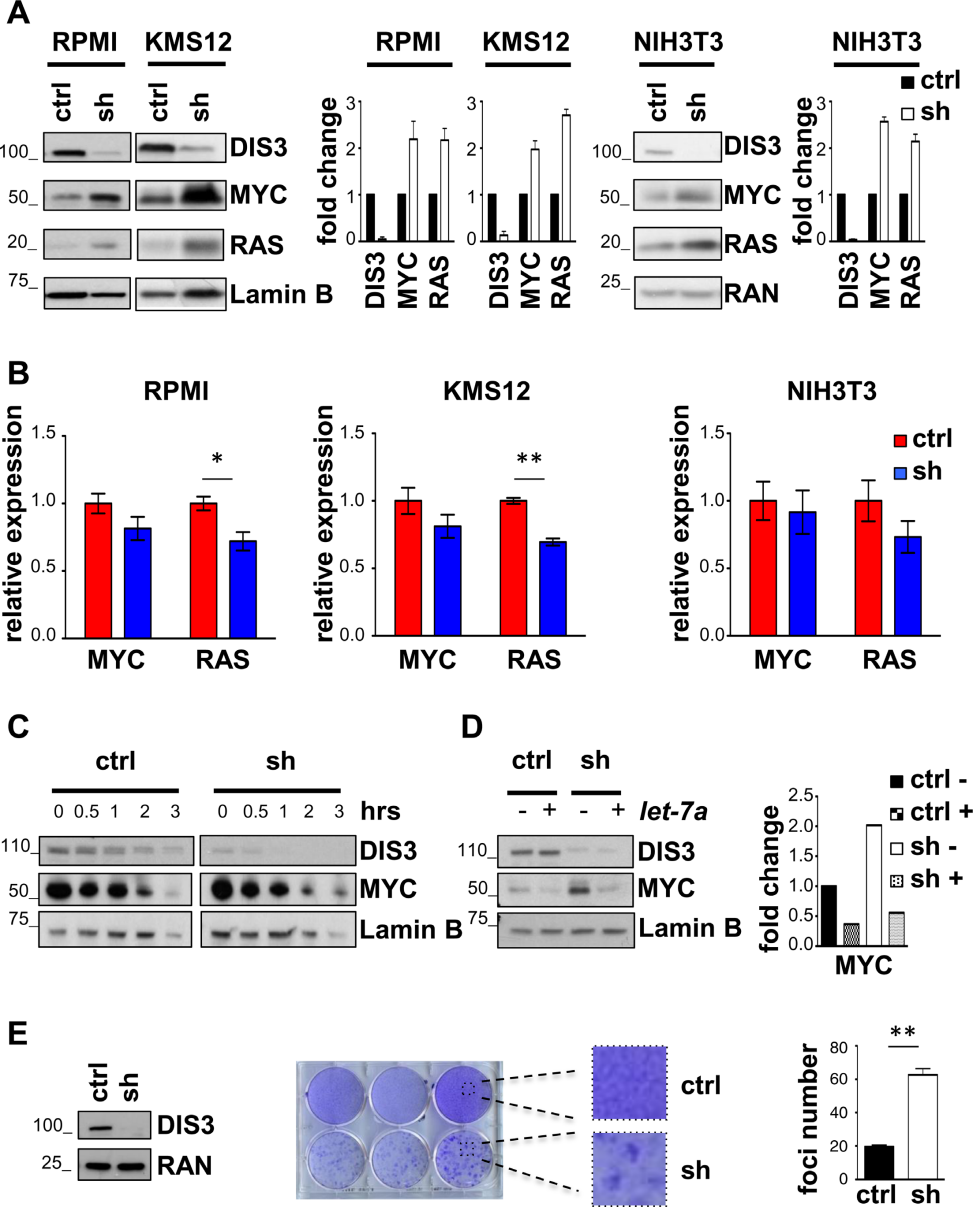
*DIS3* silencing increases MYC and RAS proteins and induces transformation. (**A**) Representative blot and quantification of DIS3, MYC and RAS proteins in RPMI, KMS12 and NIH3T3 cells, 72 h after infection with scrambled (ctrl) or human and mouse *DIS3*-specific shRNAs (sh). Results are normalized over loading control lamin B and RAN, respectively for MM cells and NIH3T3 cells. The error bars represent SD of two independent experiments. (**B**) *MYC* and *RAS* mRNA levels normalized over *GAPDH* in the same cells of panel (A). Bars represent SDs (*n* = 2 indipendent experiments). *P < 0.05; **P < 0.005. (**C**) U2OS infected with scrambled (ctrl) or *DIS3*-specific (sh) shRNAs were treated with 100 μg/ml cycloheximide for the time indicated and lysates were immunoblotted for DIS3 and MYC. Lamin B represents a loading control. (**D**) Western blot and quantification of levels for endogenous DIS3 and MYC in U2OS cells knocked-down with a scrambled shRNA (ctrl) or a *DIS3* specific shRNA (shRNA4, sh), 48 h after transfection with *let-7a* (+) or control RNA (–) mimics. Lamin B was used as loading control. (**E**) Western blot of DIS3 levels and focus formation assay of NIH3T3 cells infected with scrambled shRNA (ctrl) or with murine *DIS3* shRNA4 (sh). Colonies were counted from three independent platings. The error bars represent SD. **P < 0.005 using two-tailed Student's *t* test. RAN represents the loading control.

*Let-7* miRNAs act as tumor suppressor by reducing the levels of oncogenes including RAS, MYC and HMGA2 (24). We therefore tested the hypothesis that *let-7* reduction, mediated by DIS3 could lead to an increase in MYC and RAS levels. The depletion of DIS3 was indeed associated with an increase on MYC and RAS protein levels in MM cells as well in NIH3T3 cells and U2OS cell lines (Figure 3A and Supplementary Figure S2A). Similar results were obtained using the second shRNA, shRNA2 (Supplementary Figure S3A). We then tested whether the mRNA levels of MYC and RAS were similarly affected by DIS3 down-regulation. Notably, no significant changes in *MYC* and *RAS* mRNA levels were detected, suggesting that DIS3 controls indirectly MYC and RAS protein expression levels, not through RNA modulation (Figure 3B, Supplementary Figures S2B and S3B). To gain insight on the mechanism responsible for this phenotype, we explored the possibility that DIS3 could affect the stability of the proteins. To this end, RPMI cells were treated with cycloheximide to abolish mRNA translation, and MYC protein levels were determined by western blotting at increasing time points after the addition of the drug, with or without *DIS3* knockdown. The kinetic of MYC degradation was similar between cells infected with *DIS3* shRNA to that observed in cells infected with a control shRNA (Figure 3C). All together, these results imply that DIS3 does not affect the stability of the protein but rather promotes the translation of *MYC* mRNA. It has been previous shown that *let-7* regulate the translation of their targets, more than the mRNA levels (33–35). Indeed, our results obtained on HEK-293T cells support this notion. In these cells, where *let-7* levels did not change after *DIS3* knockdown (Figure 2), both the protein and the mRNA levels of MYC and RAS similarly remained unchanged (Supplementary Figure S4).

To conclusively demonstrate that the increase in MYC protein levels arising from *DIS3* knockdown cells was due to *let-7* down-regulation, we over-expressed a *let-7a* mimic in silenced *DIS3* cells and measured MYC levels by western blot (Figure 3D). *let-7* mimic abrogated the increase in MYC protein levels induced by DIS3 knock-down, demonstrating that DIS3 affects MYC through *let-7*.

Reduced *let-7* miRNAs correlate with increased transformation capacity *in vitro* (36). We then ascertained whether *DIS3* silencing could promote transformation. To this end, a focus formation assay was performed in NIH3T3 cells. We found that *Dis3* knockdown robustly induced focus formation, suggesting that *Dis3* silencing is able to transform fibroblasts *in vitro* (Figure 3E). This result was confirmed with a second shRNA for *DIS3* (Supplementary Figure S5).

DIS3 controls *let-7* processing through LIN28B.

**Figure 4.**
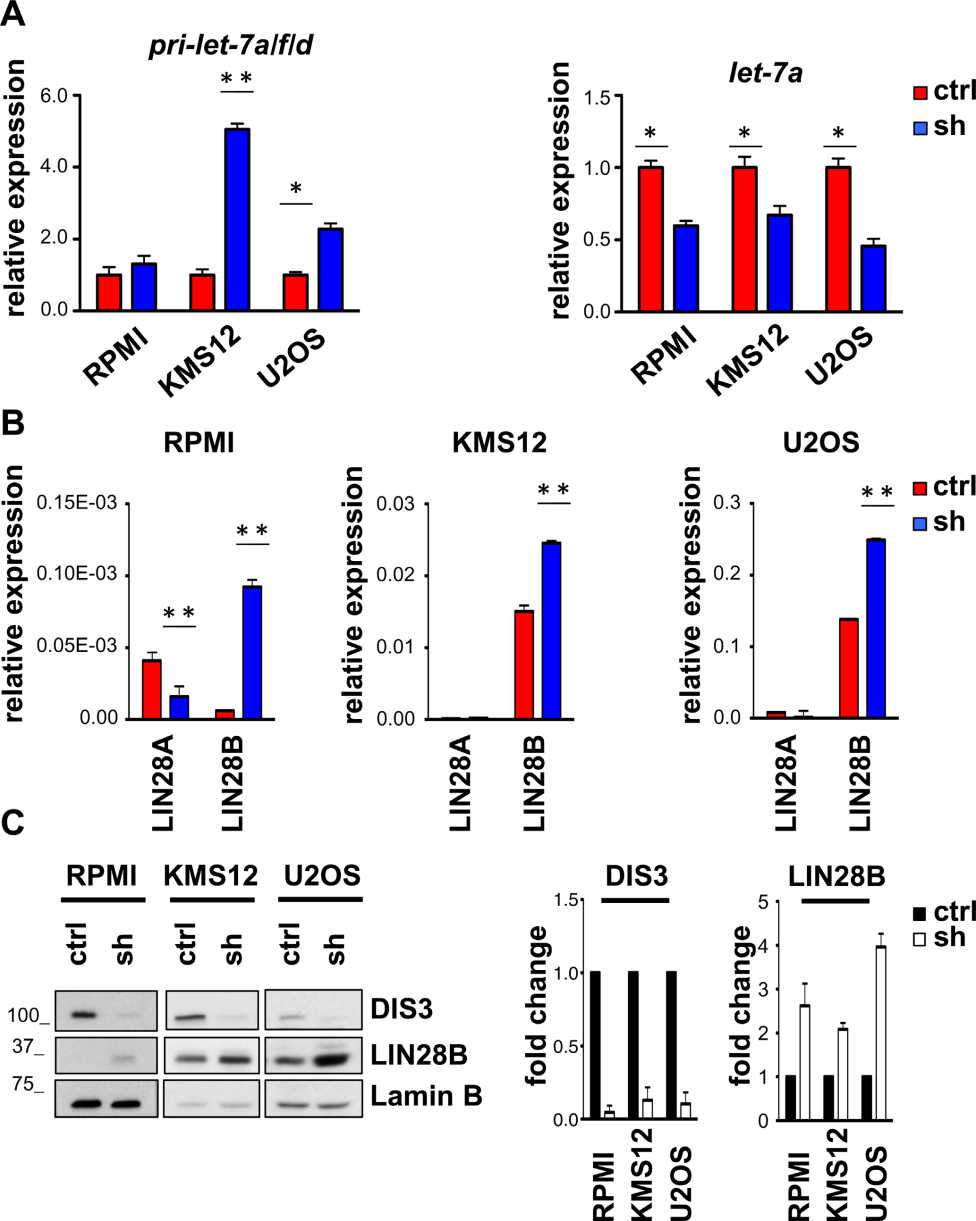
DIS3 affects *let-7* processing regulator *LIN28B*. (**A**) Measurement by qRT-PCR of the pri-miRNAs *let-7-a/f/d* (left panel) and mature miRNA *let-7-a* (right panel) in RPMI, KMS12 and U2OS cells, 72 h after infection with scrambled (ctrl) or *DIS3*-specific shRNA (sh). pri-miRNAs *let-7-a/f/d* expression data were normalized over *GAPDH*. *let-7-a* expression data were normalized over *RNU6B*. Bars represent SDs (*n* = 2 indipendent experiments). *P < 0.05; **P < 0.005 using two-tailed Student's *t* test. (**B**) *LIN28A* and *LIN28B* mRNA levels assessed by qRT-PCR with respect to *GAPDH* expression 72 h after infection. Bars represent SDs (*n* = 2 indipendent experiments). **P < 0.005 using two-tailed Student's *t* test. (**C**) Blot (left panel) and quantification (right panel) representative for two experiments showing DIS3 and LIN28B protein levels in RPMI, KMS12 and U2OS 72 h after infection with shRNA targeting *DIS3*. Lamin B was used as loading control.

Transcriptional and post-transcriptional mechanism, including processing and maturation from the pri- to pre- and mature forms, regulate miRNA levels (10). We next sought to define whether DIS3 directly affects *let-7* transcription or is instead involved in its maturation. To this end, we compared and contrasted the levels of immature pri-*let-7* with mature *let-7* after *DIS3* knockdown. We did not detect any reduction in *let-7* primary transcripts upon *DIS3* down-regulation, indeed even an increase, especially in KMS12 cells (Figure 4A, left panel). On the contrary, qPCR analysis demonstrated a consistent reduction in mature *let-7*, in *DIS3* silenced cell lines, including RPMI, KMS12 and U2OS cells (Figure 4A, right panel). The discrepancy between reduced *let-7* mature levels and unchanged or even increased *let-7* immature forms strongly suggests a role for DIS3 on *let-7* maturation. These results further imply the existence of a negative regulatory factor inhibiting *let-7* processing in *DIS3* knocked down cells.

Previous studies have demonstrated that *let-7* cleavage and maturation is under the control of the pluripotency factor LIN28 (29). We then asked whether *DIS3* down-regulation alters the levels of *LIN28A* and *LIN28B*. qPCR and western blot analysis revealed that the expression levels of *LIN28A* and *LIN28B* were variable among the cell lines tested, with KMS12 and U2OS showing the highest, and RPMI and NIH3T3 with low basal levels (Figure 4B and C and Supplementary Figures S6 and S7). Notwithstanding, we found a specific increase of LIN28B both at the RNA and at the protein level upon *DIS3* knockdown, with both shRNAs, in all cell lines where *let-7* was reduced upon *DIS3* knock-down (Figure 4B and C and Supplementary Figures S6–S8). Intriguingly, LIN28A, expressed at very low levels also in basal conditions, was not affected by *DIS3* silencing suggesting that DIS3 controls *let-7* through a selective modulation of LIN28B levels (Figure 4B). Accordingly, LIN28B levels did not change in HEK-293T cells, where *let-7* levels did not decrease upon *DIS3* knockdown (Supplementary Figure S9).

**Figure 5.**
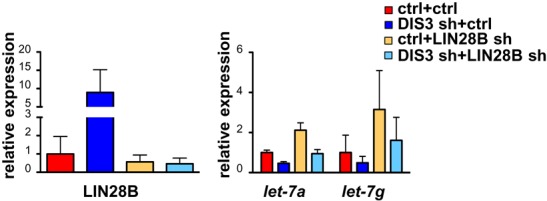
DIS3 controls *let-7* through LIN28B. *LIN28B* mRNA (left panel) and *let-7-a* and *let-7-g* (right panel) levels of one representative experiment in which RPMI cells infected with a scrambled shRNA (ctrl) or with a *DIS3* shRNA4 (DIS3 sh) underwent, after 3 days, a second infection with a scrambled shRNA (ctrl) or with a *LIN28B* shRNA. *LIN28B* and l*et-7* levels were measured 4 days after the second infection and normalized over *GAPDH* and *RNU6B* respectively. Bars represent SDs (n = 2 replicas of one representative experiment).

To conclusively demonstrate that LIN28B is required for the repression of *let-7* as a result of *DIS3* knockdown, the concomitant silencing of *LIN28B* and *DIS3* was carried out in RPMI and U2OS cells (Figure 5 and Supplementary Figures S10 and S11). We found that in both cell lines knockdown of *LIN28B* completely abrogated the reduction of mature *let-7* mediated by *DIS3* silencing, confirming that LIN28B is required for DIS3-mediated *let-7* reduction (Figure 5 and Supplementary Figure S11, right panel).

DIS3 affects RNA stability of LIN28B.

We next investigated the relationship between DIS3 and LIN28B. As DIS3 is the catalytic subunit of the exosome, a complex playing a crucial role in exosome-mediated RNA processing and decay (37) and since its silencing results in an increase of *LIN28B* mRNA levels, we speculated that DIS3 could be involved in the degradation of LIN28B RNA.

**Figure 6.**
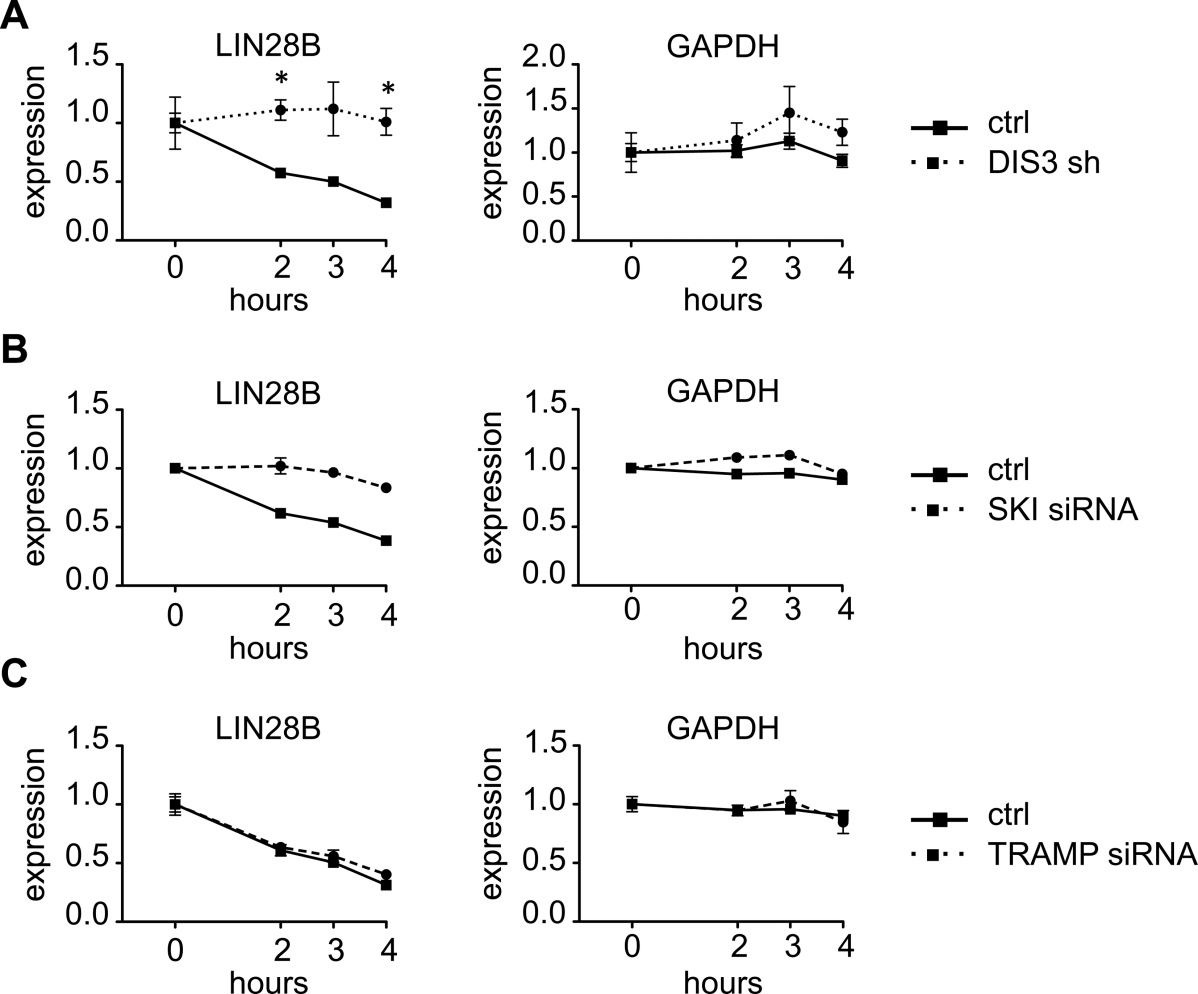
DIS3 affects RNA stability of *LIN28B* RNA. U2OS cells were infected with a scrambled shRNA (control sh) or a *DIS3* shRNA (shRNA4) (**A**) or were transfected with a control siRNA (ctrl) or a siRNA against SKI (**B**) or TRAMP (**C**). 3 days after infection and selection or 48 h after transfection, cells were treated with DRB (100 μg/ml) to block RNA synthesis. The stability of *LIN28B* and *GAPDH* total mRNA was measured by qRT-PCR at 0, 2, 3 and 4 h after treatment and expressed as relative abundance with respect to mRNA level at 0 h. Bars represent SDs (*n* = 2 indipendent experiments in A; *n* = 2 replicas of one representative experiment in B and C). *P < 0.05 using two-tailed Student's *t* test.

To test this hypothesis, we infected U2OS cells with a scrambled shRNA or with a shRNA specific for DIS3, treated cells with 5,6-dichloro-ribofuranosylbenzimidazole (DRB) to block RNA synthesis and then measured LIN28B RNA or GAPDH (as control) levels over a 4-h time interval. Upon transcriptional block, *LIN28B* RNA levels were significantly more stable after *DIS3* knockdown when compared with cells infected with scrambled shRNA (Figure 6A). On the contrary *GAPDH* mRNA half-life was not affected. These results indicate that *DIS3* specifically controls the mRNA stability of *LIN28B*.

It has been previously shown that exosome preferentially degrades RNA enriched in ARE sites (38,39). AREs consist of one or several AUUUA pentamers located in an adenosine and uridine rich region (40). These elements are bound by ARE-binding proteins endowed with RNA stabilizing or destabilizing functions, able to modulate mRNA stability or translation efficiency of mRNA target (40,41). Indeed, an analysis using the AREsite tool (http://rna.tbi.univie.ac.at/Aresite), revealed a significant enrichment of productive, accessible and conserved AU-rich elements in *LIN28B* mRNA (Supplementary Figure S12). Since mRNAs containing an AU-rich element (ARE) in the 3′ UTR undergo a rapid decay in the cytoplasm (42), we hypothesized that *LIN28B* degradation by the exosome occurs in the cytoplasm. The eukaryotic exosome associates with two ATP-dependent regulators, the cytoplasmic SKI complex and the nuclear TRAMP complex. Both interact with RNA substrates and thread them through the internal channel of the exosome core (43). Each complex is endowed with an helicase activity, provided respectively by SKI2 and MTR4, to unwind RNA targets. To determine in which cellular compartment *LIN28B* mRNA is degraded, we blocked cytoplasmic or nuclear exosome activity by transfecting U2OS with a pool of siRNAs specific for *SKI2* or *MTR4*. Forty-eight hours after transfection we treated cells with DRB and measured *LIN28B* and *GAPDH* RNA levels over a 4-h time interval (Figure 6B and C and Supplementary Figure S13). We found that the inactivation of the cytoplasmic complex SKI increased *LIN28B* mRNA stability, recapitulating the effect observed upon *DIS3* silencing (Figure 6B). Once again the stabilization was specific because no significant changes were observed on *GAPDH* mRNA half-life. On the contrary silencing of *MTR4*, the helicase included in the nuclear complex TRAMP, did not affect the stability of *LIN28B* mRNA (Figure 6C). All together these results suggest that *LIN28B* mRNA levels are specifically controlled by DIS3, mostly in the cytoplasm and probably through the recognition of ARE sites element present in the 3′ UTR.

## DISCUSSION

**Figure 7.**
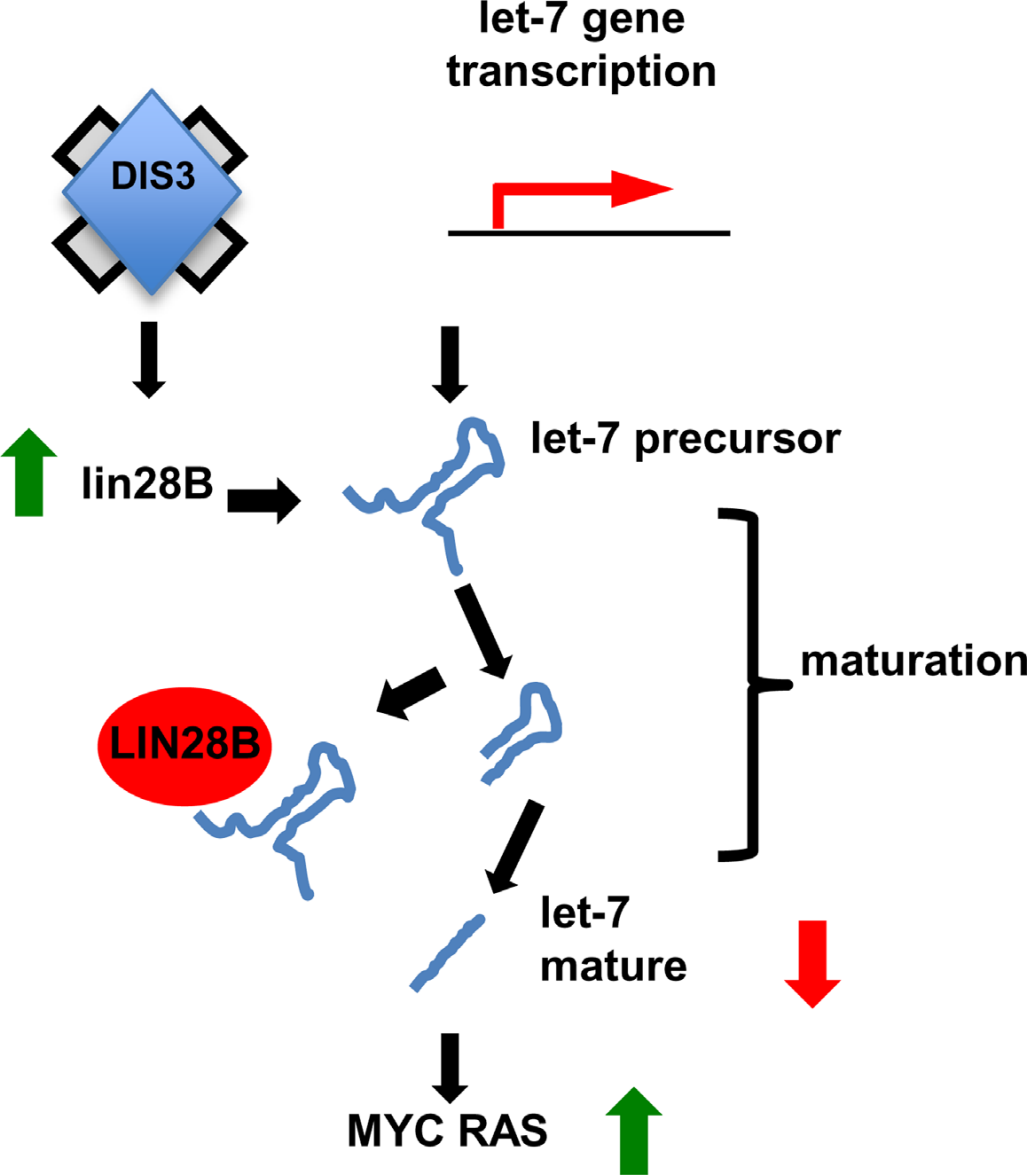
Proposed model on the role of DIS3 in regulating *LIN28B, let-7* and MYC and RAS.

In this study, we provide evidences that *DIS3* inactivation stimulates oncogenic signaling pathways through a down-regulation of the tumor suppressor miRNA family *let-7*. *DIS3* loss selectively increases *LIN28B* levels, a negative regulator of *let-7* maturation. The up-regulation of LIN28B decreases mature *let-7* and de-represses of downstream *let-7* targets. In particular the protein levels of two critical oncogenes, MYC and RAS, increased. Indeed, DIS3 knockdown transformed NIH3T3 cells (Figure 7).

Curiously, in cancer, DIS3 mutations have been found in MM and, at a lower percentage, in AML, while mutations in this gene have not been reported in the sequencing efforts ongoing in epithelial cancers, with hundreds of patient genomes screened as of today. Nonetheless, our findings suggest that DIS3 impacts on the *let-7* pathway in cell lines of hematopoietic and non-hematopoietic heritage, raising MYC and RAS protein levels in both cellular contexts. One possible explanation for the selective presence of *DIS3* somatic mutations in haematological cancers is the prominent role exerted by MYC, and to a lesser extent by RAS, in these cancers. KRAS and NRAS have been recently confirmed as the most mutated genes in MM (6,7,44,45). MYC is deeply involved in the pathogenesis of several hematological cancers. In MM, in the more advanced stages, chromosomal rearrangements juxtapose the *MYC* locus on 8q24 with the *IgH* or *IgL* locus, resulting in a several-fold enhancement of *MYC* transcription (46). Recently, a more general role for MYC has been proposed during MM pathogenesis. In fact, both mouse and human data support the notion that a moderate increase in *MYC* expression would be crucial for the transition from the pre-malignant condition of monoclonal gammopathy of undetermined significance (MGUS) toward frank MM (47). Herein, we demonstrate that *DIS3* silencing affect MYC protein levels without any change in RNA levels. It would be tempting to speculate that MM cells empower crucial oncogenes such as RAS and MYC not only increasing their mRNA levels, but, in selected cases, further boosting their activity through increased translation rate. In line with our results a recent study showed that MMSET, the histone methyltransferase translocated in up to 15% of MM patients, specifically impacts on MYC translation, and not mRNA levels, through the down-regulation of miR-126* (48). Taken together these results suggest that the dysregulation of MYC translation could have a strong impact on MM pathogenesis. As such, the conventional assessment of MYC RNA levels as a reliable readout of MYC activity might not be adequate, in particular for the identification of the patient subgroups endowed with enhanced translation.

*DIS3* is the catalytic subunit of the exosome, a multiprotein complex present both in the cytoplasm and in the nucleus that is involved in the degradation of many RNA species. The cytoplasmic exosome is not required for viability and it targets normal and aberrant mRNAs, regulating their 3′ to 5′ turnover (49,50). The activity and the role of the nuclear exosome are less well understood. Unlike its cytoplasmic counterpart, it is required for viability, and it targets a broader set of RNA species, including pre-mRNAs, pre-tRNAs, pre-rRNAs, snRNAs and snoRNAs (3,51–58). Several studies have started to comprehensively map genes and pathways affected by the specific inactivation of various exosome components (59–64). However, the mechanistic details by which the exosome or its subunits shape cell physiology are largely unknown. In particular it remains unclear whether the inactivation of the enzymatic activity of this complex would have a pervasive impact on RNAs and ultimately on cell physiology, or whether its activity is channeled through specific targets and pathways, with clear cellular outcomes. Our findings argue in favor of this second hypothesis, given the limited influence of DIS3 on miRNAs as a whole, and its specific effect on LIN28B and *let-7* family.

The human genome encodes three *DIS3* homologues: *DIS3, DIS3L*, and *DIS3L2*. Unlike DIS3 and DIS3L (65), DIS3L2 is not associated with the exosome (66,67) since it lacks the conserved N-terminal PIN and CR3 domains (65,68). Surprisingly, it has been recently shown that DIS3L2 regulates the *let-7* pathway, with a mechanism however that is entirely different from the one described herein for DIS3 (69). In fact, our data suggest that these ribonucleases regulate *let-7* biogenesis at two different steps, in two different cellular compartments and with a different mechanism. *let-7* are initially transcribed as primary transcripts (*pri-let-7*) and then cleaved by the microprocessor complex of DGR8 and DROSHA into 70- to 100-nt hairpin-shaped precursors (*pre*-*let-7*) (70). These *pre-le-7* are exported into the cytoplasm and processed by the RNase III enzyme Dicer to their mature form (*let-7*). DIS3L2 degrades uridylated pre-*let-7* in the cytoplasm, partnering with LIN28A. On the contrary DIS3 modulates *let-7* pathway through the regulation of LIN28B levels, thus affecting the abundance of primary *let-7* transcripts available for microprocessor activity. Therefore, the inactivation of DIS3L2, as reported in Perlman syndrome and in a subset of Wilms tumors, would have entirely different functional consequences for the cell, when compared with the effect of DIS3. In fact, *DIS3L2* knockdown results in the accumulation of uridylated *pre-let-7* with no changes in the levels of mature *let-7*, consistent with the DIS3L2 preferential targets, pre-*let* previously modified (uridylated) and thus no more available for maturation. On the contrary *DIS3* silencing reduces *let-7* mature levels, ultimately affecting the whole *let-7* pathway downstream, an outcome not anticipated in the presence of *DIS3L2* inactivation.

In conclusion, we reveal a novel regulation pathway of *let-7* miRNAs controlled by the exoribonuclease DIS3. We identified LIN28B as a direct target of the enzymatic activity of DIS3 and we demonstrated that through LIN28B DIS3 selectively regulates a subset of critical *let-7* targets such as MYC and RAS. Future studies are warranted to determine the extent to which this pathway is affected in MM patients mutated for DIS3 and how it contributes to MM pathogenesis.

## SUPPLEMENTARY DATA

Supplementary Data are available at NAR Online.

SUPPLEMENTARY DATA
