# Supplementary material for: The ribonuclease DIS3 promotes let-7 miRNA maturation by degrading the pluripotency factor LIN28B mRNA
Source: Nucleic Acids Res. 2015 Apr 29;43(10):5182–93. doi: 10.1093/nar/gkv387 (PMC4446438; doi:10.1093/nar/gkv387)
Supplement: SUPPLEMENTARY DATA [file supp_43_10_5182__index.html]

The ribonuclease DIS3 promotes let-7 miRNA maturation by degrading the pluripotency factor LIN28B mRNA — The ribonuclease DIS3 promotes let-7 miRNA maturation by degrading the pluripotency factor LIN28B mRNA — The ribonuclease DIS3 promotes let-7 miRNA maturation by degrading the pluripotency factor LIN28B mRNA — SUPPLEMENTARY DATA 

# The ribonuclease DIS3 promotes *let-7* miRNA maturation by degrading the pluripotency factor *LIN28B* mRNA

## SUPPLEMENTARY DATA

**Files in this Data Supplement:**

- SUPPLEMENTARY DATA
