# Supplementary material for: The ribonuclease DIS3 promotes let-7 miRNA maturation by degrading the pluripotency factor LIN28B mRNA
Source: Nucleic Acids Res. 2015 Apr 29;43(10):5182–93. doi: 10.1093/nar/gkv387 (PMC4446438; doi:10.1093/nar/gkv387)
Supplement: SUPPLEMENTARY DATA [file supp_gkv387_nar-02759-a-2014-File009.pdf]

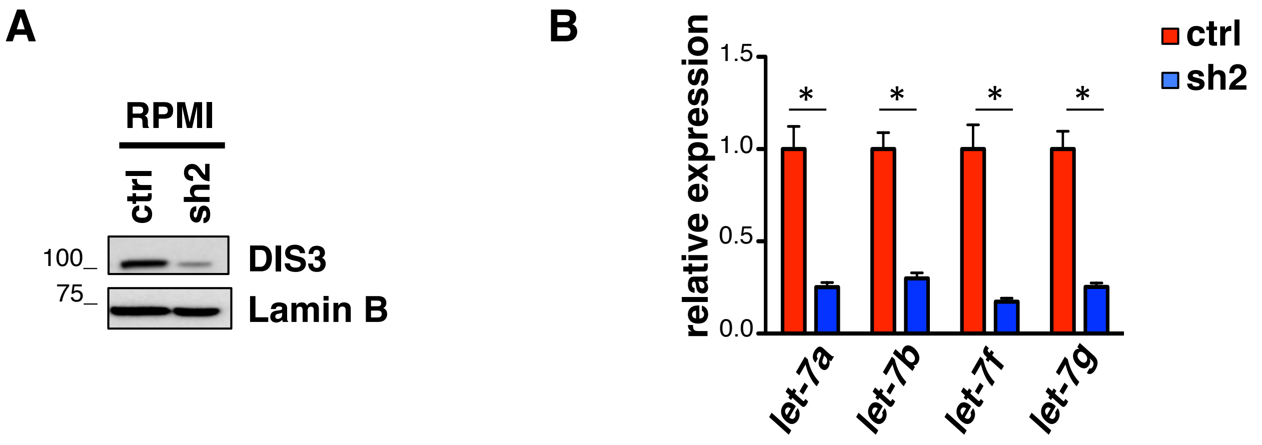
A
RPMI
ctrl
sh2
100_
75_
DIS3
Lamin B
B
relative expression
1.5
1.0
0.5
0.0
*
*
*
*
let-7a
let-7b
let-7f
let-7g
ctrl
sh2

Suppl. Fig.1

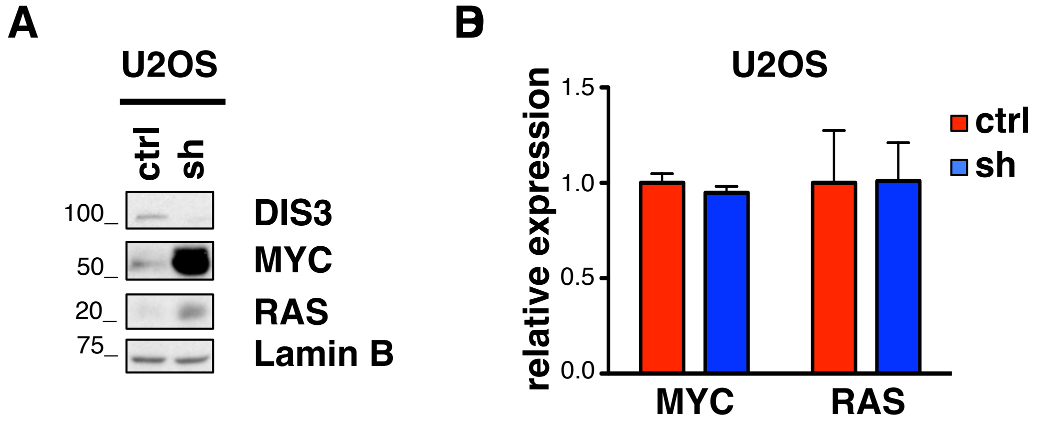
A
U2OS
ctrl
sh
100_
DIS3
50_
MYC
20_
RAS
75_
Lamin B
B
U2OS
relative expression
1.5
1.0
0.5
0.0
ctrl
sh
MYC
RAS

Suppl. Fig. 2

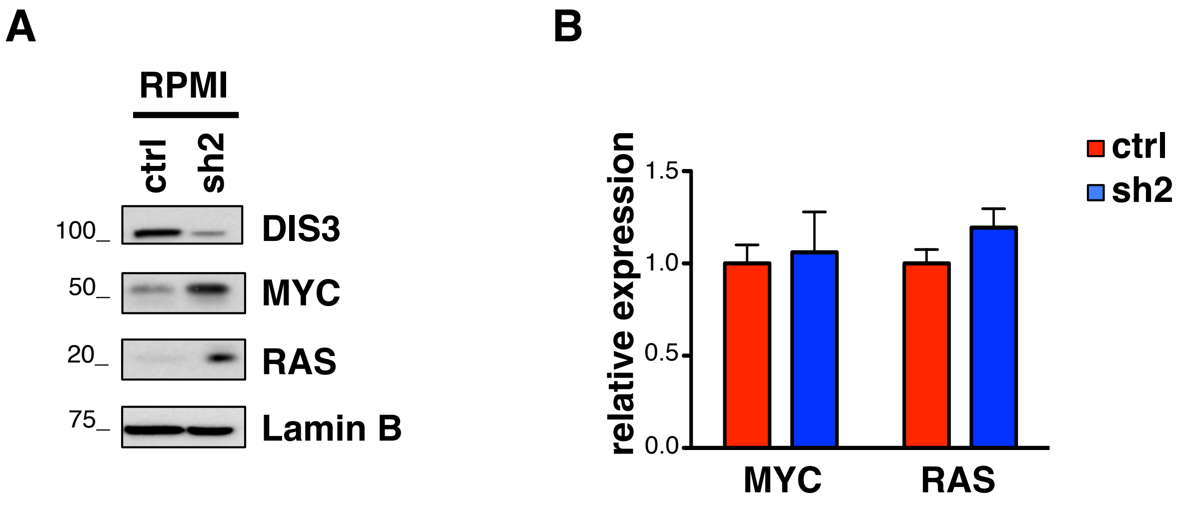
A
RPMI
ctrl
sh2
100_
DIS3
50_
MYC
20_
RAS
75_
Lamin B
B
relative expression
1.5
1.0
0.5
0.0
MYC
RAS
ctrl
sh2

Suppl. Fig. 3

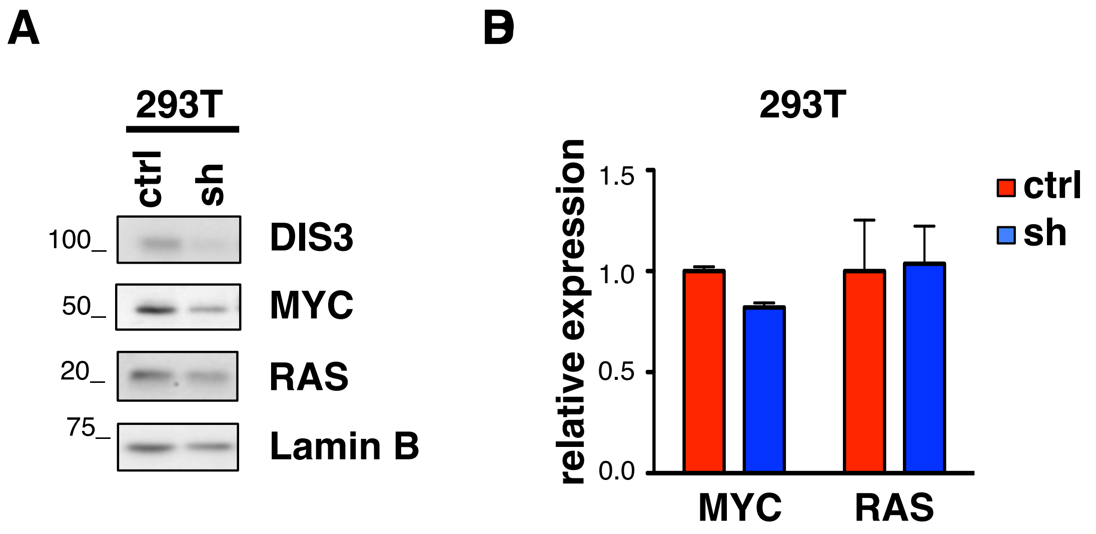

A
293T
ctrl
sh
100_
DIS3
50_
MYC
20_
RAS
75_
Lamin B
B
293T
relative expression
1.5
1.0
0.5
0.0
ctrl
sh
MYC
RAS

Suppl. Fig.4

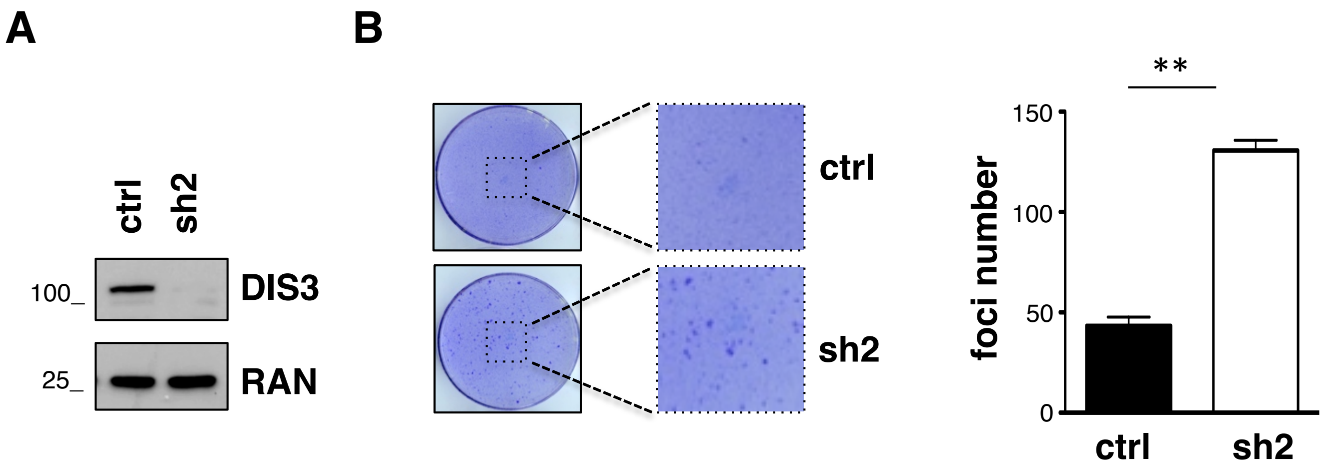
A
ctrl
sh2
100_
DIS3
25_
RAN
B
ctrl
sh2
**
150
100
50
0
foci number
ctrl
sh2

Suppl. Fig. 5

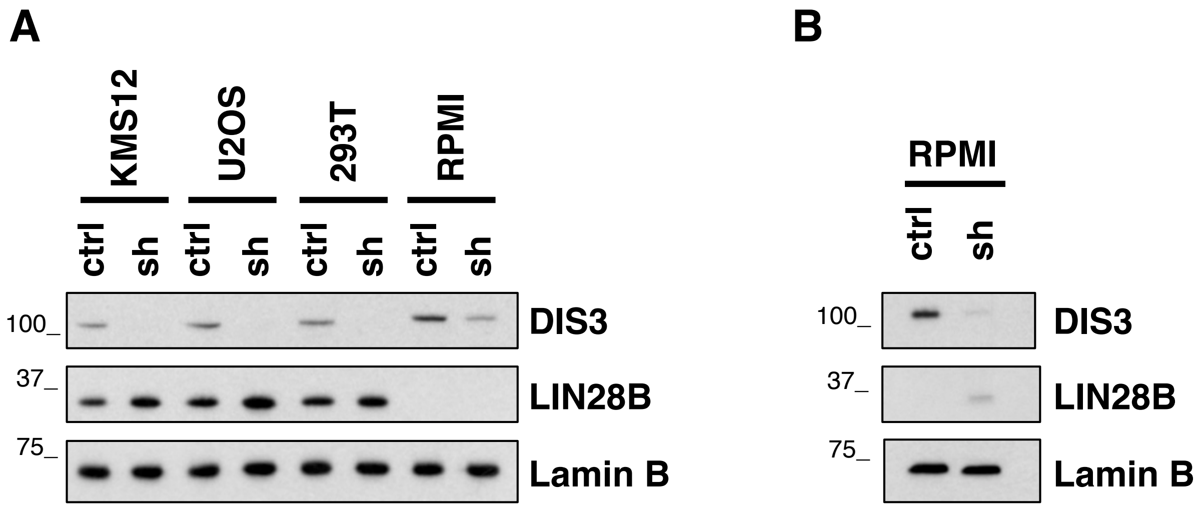
A
KMS12
U2OS
293T
RPMI
ctrl
sh
ctrl
sh
ctrl
sh
ctrl
sh
100_
37_
75_
DIS3
LIN28B
Lamin B
B
RPMI
ctrl
sh
100_
37_
75_
DIS3
LIN28B
Lamin B

Suppl. Fig. 6

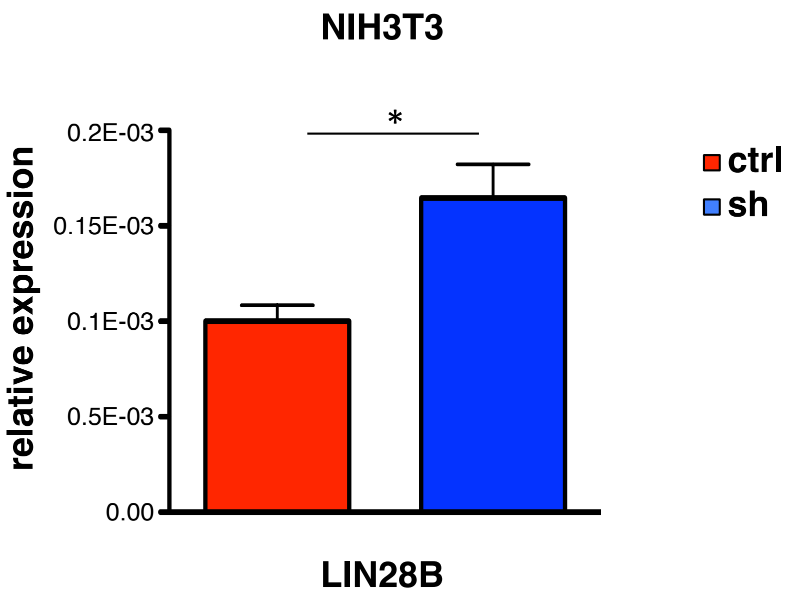
NIH3T3
*
0.2E-03
0.15E-03
0.1E-03
0.5E-03
0.00
relative expression
ctrl
sh
LIN28B

Suppl. Fig.7

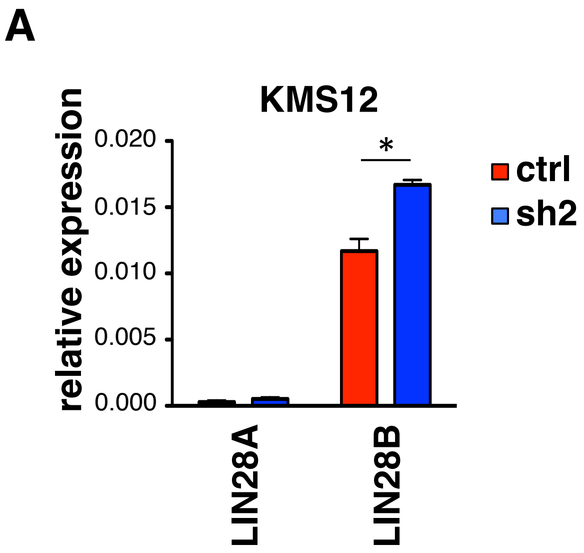
A
KMS12
relative expression
0.020
0.015
0.010
0.005
0.000
*
ctrl
sh2
LIN28A
LIN28B

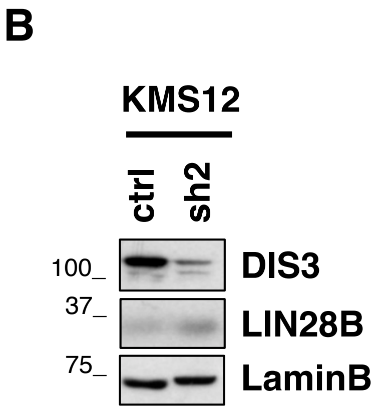
B
KMS12
ctrl
sh2
100_
37_
75_
DIS3
LIN28B
LaminB

Suppl. Fig. 8

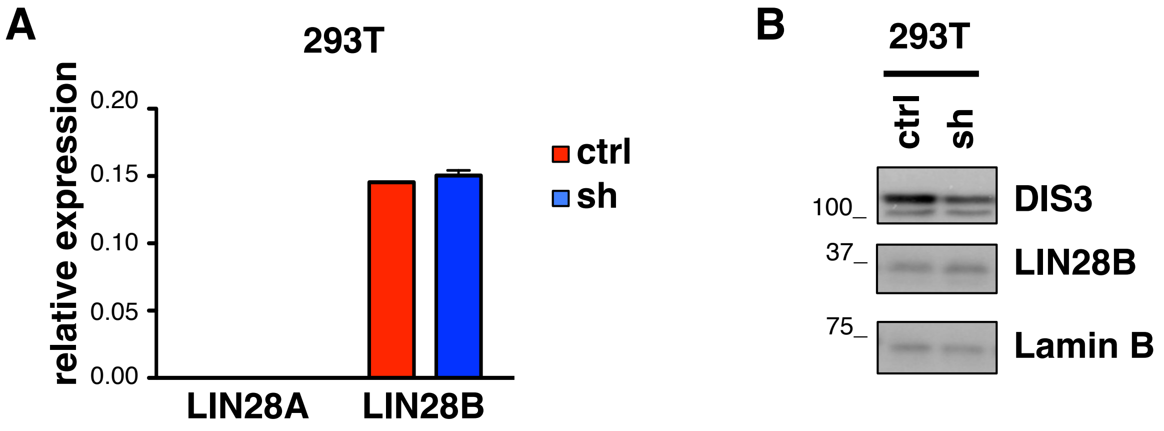
A
293T
relative expression
0.20
0.15
0.10
0.05
0.00
LIN28A
LIN28B
ctrl
sh
B
293T
ctrl
sh
100_
DIS3
37_
LIN28B
75_
Lamin B

Suppl. Fig. 9

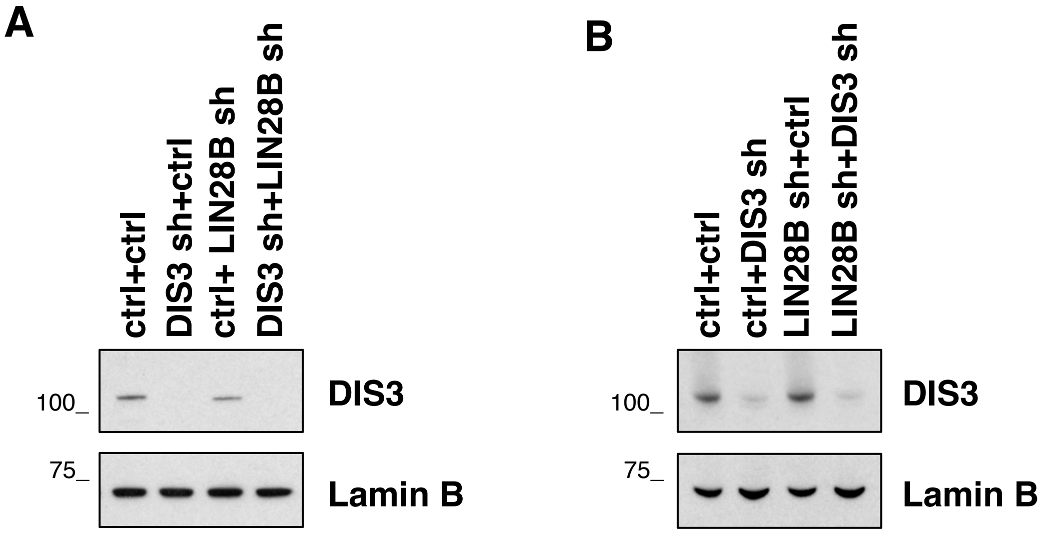
A
ctrl+ctrl
DIS3 sh+ctrl
ctrl+ LIN28B sh
DIS3 sh+LIN28B sh
100_
75_
DIS3
Lamin B
B
ctrl+ctrl
ctrl+DIS3 sh
LIN28B sh+ctrl
LIN28B sh+DIS3 sh
100_
75_
DIS3
Lamin B

Suppl. Fig.10

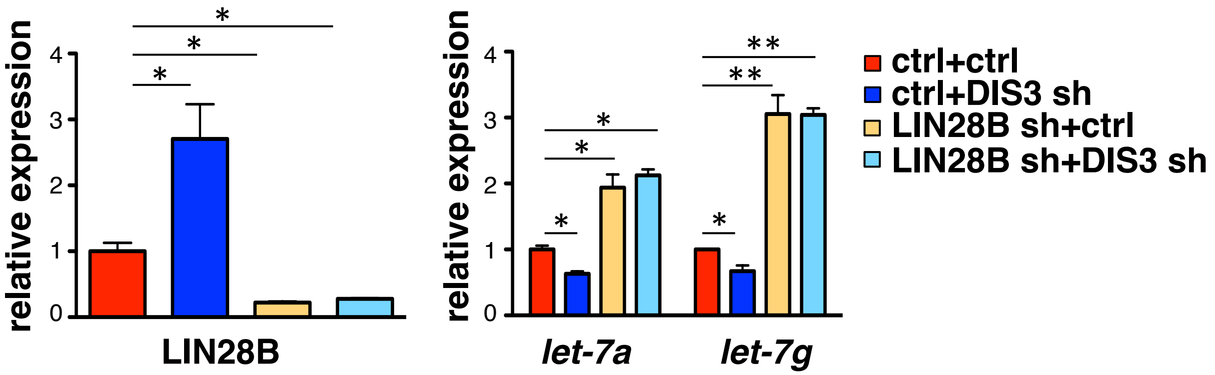
relative expression
4
3
2
1
0
*
*
*
LIN28B
relative expression
4
3
2
1
0
*
*
*
**
**
*
let-7a
let-7g
ctrl+ctrl
ctrl+DIS3 sh
LIN28B sh+ctrl
LIN28B sh+DIS3 sh

Suppl. Fig. 11

A

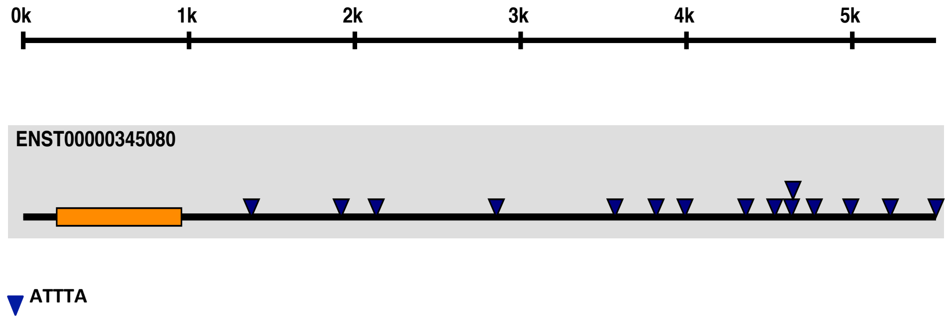

B

Transcript ENST00000345080 (representative transcript)

**Length 3' UTR:**	4,549 nt
**A+T content in 3' UTR:**	0.64
**RNAplfold output:**	[opening energies | probabilities of being unpaired] (whole transcript)
**Download/Linkout:**	[download as annotated Genbank file | Linkout to e!Ensembl]
**ATTTA:**	**0.94** (mononucelotide) / **98.52** (dinucleotide) fold-enrichment
**Site 1371-1375:**	ATTTA (ATTTA)<br>Opening energy for the core AUUUA pentamer: **0.37** kcal/mol (short range) / **0.72** kcal/mol (mid range)<br>Probability of being unpaired for the core AUUUA pentamer: **0.55** (short range) / **0.31** (mid range)<br>[Highlight | show accessibility plot | show sequence logo | show alignment]
**Site 1914-1918:**	ATTTA (ATTTA)<br>Opening energy for the core AUUUA pentamer: **0.23** kcal/mol (short range) / **0.89** kcal/mol (mid range)<br>Probability of being unpaired for the core AUUUA pentamer: **0.69** (short range) / **0.24** (mid range)<br>[Highlight | show accessibility plot | show sequence logo | show alignment]

Site 2124-2128: ATTTA (ATTTA)

Opening energy for the core AUUUA pentamer: **0.62** kcal/mol (short range) / **1.13** kcal/mol (mid range)

Probability of being unpaired for the core AUUUA pentamer: **0.37** (short range) / **0.16** (mid range)

[Highlight | show accessibility plot | show sequence logo | show alignment]

Site 2849-2853: ATTTA (ATTTA)

Opening energy for the core AUUUA pentamer: **0.29** kcal/mol (short range) / **0.96** kcal/mol (mid range)

Probability of being unpaired for the core AUUUA pentamer: **0.62** (short range) / **0.21** (mid range)

[Highlight | show accessibility plot | show sequence logo | show alignment]

Site 3569-3573: ATTTA (ATTTA)

Opening energy for the core AUUUA pentamer: **1.10** kcal/mol (short range) / **1.46** kcal/mol (mid range)

Probability of being unpaired for the core AUUUA pentamer: **0.17** (short range) / **0.09** (mid range)

[Highlight | show accessibility plot | show sequence logo | show alignment]

Site 3812-3816: ATTTA (ATTTA)

Opening energy for the core AUUUA pentamer: **1.05** kcal/mol (short range) / **1.57** kcal/mol (mid range)

Probability of being unpaired for the core AUUUA pentamer: **0.18** (short range) / **0.08** (mid range)

[Highlight | show accessibility plot | show sequence logo | show alignment]

Site 3986-3990: ATTTA (ATTTA)

Opening energy for the core AUUUA pentamer: **0.85** kcal/mol (short range) / **0.34** kcal/mol (mid range)

Probability of being unpaired for the core AUUUA pentamer: **0.25** (short range) / **0.58** (mid range)

[Highlight | show accessibility plot | show sequence logo | show alignment]

Site 4355-4359: ATTTA (ATTTA)

Opening energy for the core AUUUA pentamer: **1.62** kcal/mol (short range) / **2.12** kcal/mol (mid range)

Probability of being unpaired for the core AUUUA pentamer: **0.07** (short range) / **0.03** (mid range)

[Highlight | show accessibility plot | show sequence logo | show alignment]

Site 4529-4533: ATTTA (ATTTA)

Opening energy for the core AUUUA pentamer: **1.31** kcal/mol (short range) / **2.08** kcal/mol (mid range)

Probability of being unpaired for the core AUUUA pentamer: **0.12** (short range) / **0.03** (mid range)

[Highlight | show accessibility plot | show sequence logo | show alignment]

Site 4633-4637: ATTTA (ATTTA)

Opening energy for the core AUUUA pentamer: **1.16** kcal/mol (short range) / **1.23** kcal/mol (mid range)

Probability of being unpaired for the core AUUUA pentamer: **0.15** (short range) / **0.14** (mid range)

[Highlight | show accessibility plot | show sequence logo | show alignment]

Site 4643-4647: ATTTA (ATTTA)

Opening energy for the core AUUUA pentamer: **1.09** kcal/mol (short range) / **1.40** kcal/mol (mid range)

Probability of being unpaired for the core AUUUA pentamer: **0.17** (short range) / **0.10** (mid range)

[Highlight | show accessibility plot | show sequence logo | show alignment]

Site 4773-4777: ATTTA (ATTTA)

Opening energy for the core AUUUA pentamer: **1.44** kcal/mol (short range) / **1.33** kcal/mol (mid range)

Probability of being unpaired for the core AUUUA pentamer: **0.10** (short range) / **0.11** (mid range)

[Highlight | show accessibility plot | show sequence logo | show alignment]

Site 4987-4991: ATTTA (ATTTA)

Opening energy for the core AUUUA pentamer: **1.07** kcal/mol (short range) / **2.08** kcal/mol (mid range)

Probability of being unpaired for the core AUUUA pentamer: **0.18** (short range) / **0.03** (mid range)

[Highlight | show accessibility plot | show sequence logo | show alignment]

Site 5225-5229: ATTTA (ATTTA)

Opening energy for the core AUUUA pentamer: **0.58** kcal/mol (short range) / **0.57** kcal/mol (mid range)

Probability of being unpaired for the core AUUUA pentamer: **0.39** (short range) / **0.39** (mid range)

[Highlight | show accessibility plot | show sequence logo | show alignment]

Site 5501-5505: ATTTA (ATTTA)

Opening energy for the core AUUUA pentamer: **0.21** kcal/mol (short range) / **1.57** kcal/mol (mid range)

Probability of being unpaired for the core AUUUA pentamer: **0.71** (short range) / **0.08** (mid range)

[Highlight | show accessibility plot | show sequence logo | show alignment]

C

3' UTR sequence

CAGGTCTTCTTCATATGTTCTTTCCTTTACCCGGTTGCAAAGTCTACCTCATGCAAGTATAGGGGAACAGTATTTCACAA
GCAGTAGCTGACCTGGGATTTTAACTACTATTGGGGAACTGTGAATTTTTTAAACAGACAAATCACTCTAAGCAAATTAC
ATTTGAGCAGGGTGTCATGTTTTATGTTAATTCAGAGAATAAGATACTATGTCTGTCAATATGTGCATGTGTGAGAGGGA
GAGAGCCTGAGTCTGTGTGTGTACATGAGGATTTTTATATAGGAATGTAGACACATATATAAAGAGGCTTTGTCTTTATA
TATTTGTGTATAGATCAAAGCACACACCCTCTCTCATATAATTGGATATTTCCAAGAATTGAAAACCCATGTGAAGCATT
ATAGATAGTTTTAAATTTAACCCACTGGAGTTTTCTTGAAATACCACTTCTTTTATATTATATAAAACTAAAAACACGAC
TGTTACCTTTTGTGTGAACCAAAGGATACTTCAGATCTCAGAGCTGCCAATTATGGGGTACTAAAGGTTTTTAAGACATC
CAGTTCTCCCGAATTTGGGATTGCCTCTTTTTCTTGAAATCTCTGGAGTAGTAATTTTTTTCCCCCTTTTTTGAAGGCAG
TACCTTAACTTCATATGCCTCTGACTGCCATAAGCTTTTTTGATTCTGGGATAACATAACTCCAGAAAAGACAATGAATG
TGTAATTTGGGCCGATATTTCACTGTTTTAAATTCTGTGTTTAATTGTAAAATTAGATGCCTATTAAGAGAAATGAAGGG
GAGGATCATCTTAGTGGCTTGTTTTCAGTAGTATTTTAATATCAGCTTCTTGTAACCTTTTCCATGTTGTGAGGGTTGTA
AGGGATTGTGTGGCAACAGCAGCTTCCCTTGGCTAACTCAATCTTCTACCCATTGCTTAGAGCAGGGAGCCCTCCTTATT
TACTACTGAAGACCTTAGAGAACTCCAATTGTTTGGCATATATTTTTGGTGGTGGTTTTTATTCCTCCTGGAGAGTTATC
TAATTTGTTTCTAAAACAAACAAGCAGCAAAGAAATGAATTAAATACTGGGGTTGAGAATTAAAATTAAGTGGATGTTCA
CAGTTGCCCAATATATATGACCTGCAAATGATACGAAAAAGTGCAGCATTTAGTGGCAGTTAACAAGAGTGACAAGCCTG
GGGCAGAGGTACCAAACCTCTCCCACCAGAGAGCTAGAAGTATTTTATACAGTAACTTTGATCTTATGGAAGTGACCTTC
AATGCTTATTCTGAAGTAACCTATATGGTGGATACAGGATGAACATTCAGTGCCAGGGAGAATCTTCTCAGGTTGGTTCT
CGTTAGAGTGATAAACTGGCTAGGGGCCATAGTATTGGTCCTGTTAGGTTTCGGTCATGGAAAAAAAAATTATTTTGGGG

TCATCCTGGCTCTAGATGTTATGGGCAAATTTCTGAAACATCTGCAAGAAGGTACCAGTTAATTATAGTGCTTAATATTG
GGAATAAGATTAAGCATTATAATTATAATGTATGGGCCTGTTGGTGTAAGCTCAGATAATTAAATAAAAATAGCATGACT
CAAATGAGACATATTCTGCTGAACAGTTTCTACTTCCTCTCCCGCCTGTCCTGTCATGGGAGACGTGTATAGTTGCTGCT
GTTTCAGCAAACCACCATAAGACGAAAATGCCTCAGGTTGGGTTGCCAGTCCTTTACAACTCAGCTTGAATTTCACAACA
GTGATTGTGAGAATCTGCGTGGTATACACTGAAATATCGGTGTGCTGTGATGCAAAGCTTACCTTTGACGATATTGAATG
TGATATAGCTGTAGAGAAGTACTTCCTTGCCTTATGTGAGGATTTCAAACTTATTTAAATTATGTAGACAAATCAAAGTG
GCATTGCTTAATTTTTAGCAGGCATAATAAGCAAGTTAACAGTAAAATGCAAAACATGATAAGCGTTGCTCAATTTTTAG
CAGGTATAATAAGCAGGTTAACAGTAAAAATGCAAAACATGATAGATAAGTCACTTTGAAAATTCAAACCAAAGTTCCTT
CACCTTATGGAAATAGGAAATTATGGACTTCAAAATTGGACACTTCCTGTTTACAAAAAGAAATTCAGAGCTAAAATCAT
GGTAAAAAAAAATAGAAACACTTGAGAACTATGGTCTTTATGGGTGCAATTTGAAATCCTTTTCATCATCTTACCAGACT
AAACTAAGAGCACATACCAAACCTATCTTATGGTTGAAAGTTGGGGTTTATTTTTTATATGAGAATATTATCACTATTAC
ATAACATACTCAGGACAAAGAACTTTGCTCAGGGAACATACCATGTAATATTTTTGTTGTTTCTTTACAGACTAGTCTAC
AGTCCTGCTTACTCAAAACAAACCAAATAACTTATACCTTTATATAAGTATTATGTACTGATGATAGTAACTACCTCTGA
GTTTGACACAGATCAAAATTTTTGAATATCAGATATCAGTTATCCTATTTTTATTTCATGTGAAAACTCCTCTAAAGCAG
ATTCCCTCAACTCTGTGCATATGTGAATATCACTGATGTGAACACATTGTTCATTTACATAGGTAAAATATTACTCTGTT
TACAGCAAAAGGCTACCTCATAGTTGATACATAGCACACCTGTATGTATGCTGTTCCAGCCTTACAGGTGGCTGATAATT
CTCTGGTACAGAACCTTTTTATCTGTATTATAAATAGCAATTCACAACTGCATGTTTCTGACAAACACTTGTGAATAATG
AAGCATCTCGTTTTAGTTAGCAAAGTCTCCAAACATTTCCTTAAAATAATCATGTATTTAGTTTAAAGAATTATGGGCAC
TGTTCAACTTAAGCAAAACAGAACACGGAAGCAGTCTTAGAAGCACCACTTTGCCCAGAGGTGGAGGTTGGAAGGGGTAG
CAGGGAGAGGGGTTGGTGTATGCAGGTATTCATGCTAGGCAAAGAGTTTAAAAGACGCCAATGTCCTTCATTTACTGTCT
GTGCTGCCCTGAAGCCAAGCGTATTGCAGCATTATAGCCCCAGGCACATAACTAACTAGCACTGGCTTGCCAAGGAATGA
ACATGCAATGCCATTACTAGCTATTGAGGGAAAAGGGTCTGTGTGAAGCATCACTTTGCAGGGATTACTAATGGTGGGGC
AGCAGGTCTGTGAATTAAGTTATCTCTTGACCTCACCCTCATGTCAACACAAATGTAATTCCTAAACAAGATGCATTGCC
AGTCTCTTAGCCCTGTAAGCTGATCTTTTGCTACATGGCAGACTATAATGAAAACATTTTTATACTTGGGTTTCTAGTCT
TCACTAGAAGGCCTTGGATGTATTTTTGCAGTTGAAAGATTTAGAAAGATTTTTACCTGCTTATAACTTGGAAGTTTAGA
GTGCAATGTAAGAAAAAAGATCAAGAAATGTCATGTTATTAGCATCAGTCCACCTCCAATATTGCCGATACTTTTTTTAT
TCTGGCTCAGTTTTATTTTGCACCAGTGCGGCCCCAAGTTACTGCTGGTTGTATTTAGTTTGTGAATAGGAGCCCATAAG
TGTTAATAGACTTTGTAACATTCACTATAAGATGAATTATACAGGACATGGGAAATCTCATTAAGTCTTAAAGTTAATTT
AAATTAATTTATCTGTTTTCTCTAAGAAATGTTTATCATAAAATATATATGTGTATTTCCCCTTTGGTTATAAAATTTGG
GAAAGTATGTACAAGTGCAGCTGCACTGACTTTAATTTTCTAGATGTCTTAATGAGATTTATTTGTTTTAGAGAAGAACA
TCTTGTTAAAAGCATCAAACTCTGTCTTACATAGCTGTCAACAGCCTCTTTAAGATGTGGTGGTTGTATGATCTGTGTCT
TAATTGTTCAGTTAGAGTGAGAAGTTGACCTATGATTCATTTTTAAATTTTATATTTGGAACAAAGCTGCAAGTTATGGT
AAAGTACTGTACTGTGAGAAGTATTATGATATTTAATGCATCTGTGGCTTAACACTTGTGAGAGTTACCAGCTTGAAAAT
GATGGTGTTGACTACCTCTTGAATCACATCTATCAACCACTGGCACCTACCACCAAGCTGGCTTCAATTAGTATGTGTTG
CTTTTTGGTATTAACAACTAACCGTACTAGAGACCAAAGTGAACCCTGATTTTTATATGTCTTTAATAATGGTGTTTTAT
CTAGTGTTTTTAAATTATCCTGTGTAGTATTTAGATTACCTCATTGTCCATTTTGACTCATGTTGTTTACAAGTGAAAAT
AAAAACACTTGAACTGTATGTTTTTAAAAGACAAAAAAGGGGTAGATGTTTGGAATGCGTTTCACTCGCATGCAGTCATC
TGGAGGGACTGAAGCACTGTTTGCCTTTCTGTACACTCTGGGTTTTATATTCTCATTTCATGCCTAATGTCTTATTCTGT
CAATTATGGATATGTTGAGGTTTAAAAAAATTACTTGATTAAAAATAAAACATATAACGTTGGCATTTA

D

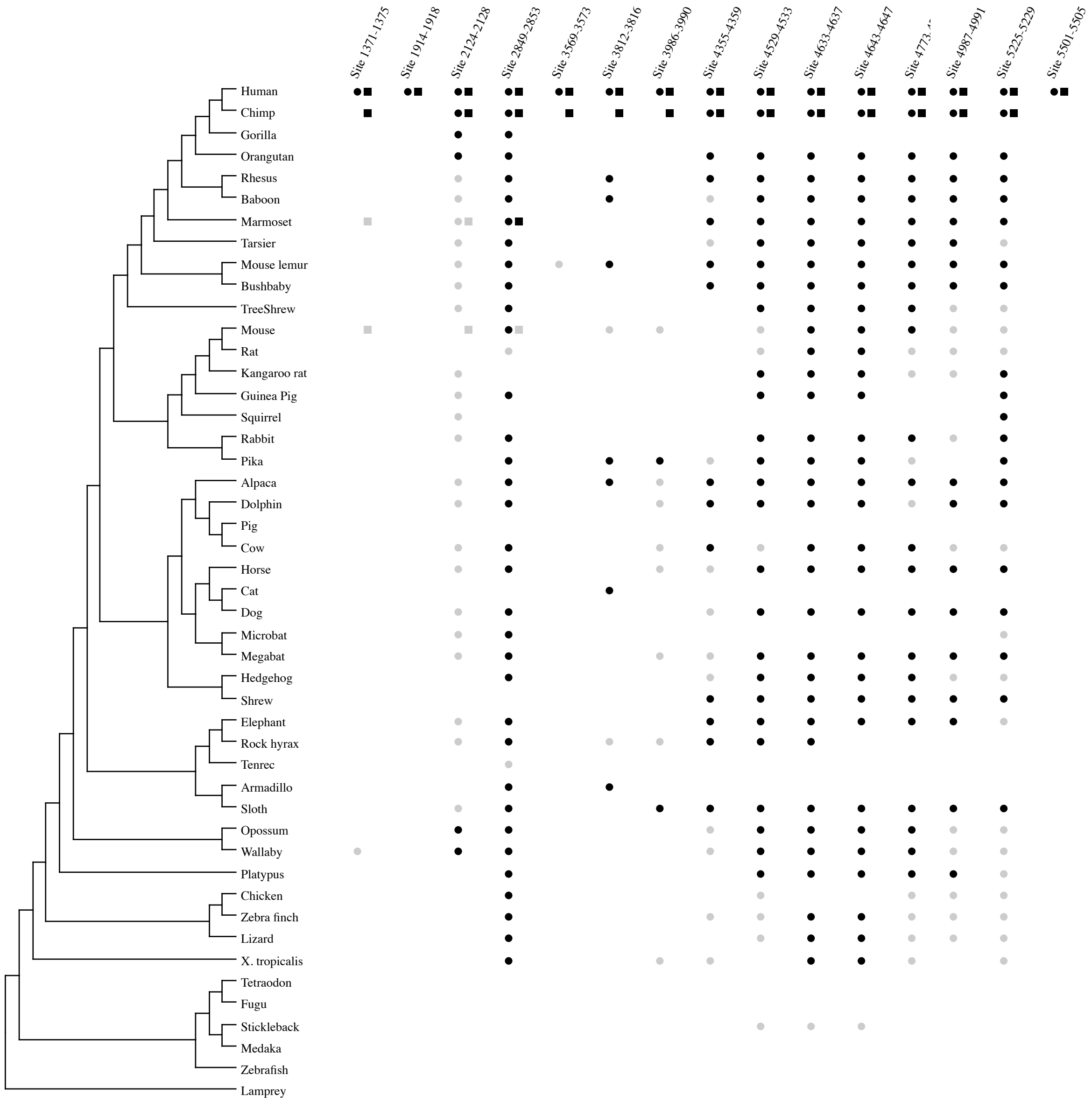

Suppl. Fig 12

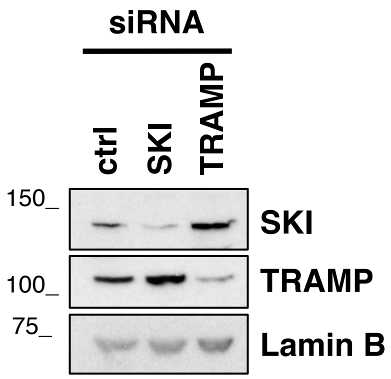

Suppl. Fig. 13

Table S1. The short hairpin RNA sequences inserted in pLKO.1 non inducible lentiviral plasmid.

Name	Species	sequence	Company
DIS3 sh1	H. Sapiens	AATAGCAACAATAATTCTCCG	Open Biosystem
DIS3 sh2	H. Sapiens	ATTACATTCCTGATGGCAGGG	Open Biosystem
DIS3 sh3	H. Sapiens	ATATCAGTACATCCTGGTGGG	Open Biosystem
DIS3 sh4	H. Sapiens	AAGACGATCTATGAGTTCGGG	Open Biosystem
DIS3 sh5	H. Sapiens	AAATGTGTGTATATTGGAGAC	Open Biosystem
DIS3 msh	M. Musculus	AATTCTCCGTCCTTCTAACGC	Open Biosystem
DIS3 msh2	M. Musculus	AATAGCGACAATAATTCTCCG	Open Biosystem
LIN28B sh	H. Sapiens	GCCTTGAGTCAATACGGGTAA	Open Biosystem
Control sh	None	CAACAAGATGAAGAGCACCAA	Sigma Aldrich

Table S2. Antibodies used in western blot assay.

Protein	Company	Catalog number	Dilution
DIS3	ProteinTech	#14689-1-AP	1:1000
H-Ras	Santa Cruz	#sc-520	1:500
c-MYC	Cell Signaling	#9402	1:1000
LIN28B	Cell Signaling	#4196	1:20000
LaminB	Santa Cruz	#sc-6216	1:1000
RAN	Becton Dickinson	#610340	1:75000
SKI2	Duotech	11462-1-AP	1:500
MTR4	Abcam	AB70551	1:2000

Table S3. Primers used in qRT-PCR

Gene	Species	Sense primer	Antisense primer
MYC	H. Sapiens	TCAAGAGGCGAACACACAAC	GGCCTTTTCATTGTTTTCCA
MYC	M. Musculus	GTGCTGCATGAGGAGACACC	GCCTCTTCTCCACAGACACC
RAS	H. Sapiens	AGCCCCAGCTCAGCGGATGAA	CTGCCGTCTACTCCTCTTGGAT
RAS	M. Musculus	CCTTCGAGGACATCCATCAG	CTGAATCTTTCACCCGCTTG
GAPDH	H. Sapiens	AGCCACATCGCTCAGACAC	AGCCACATCGCTCAGACAC
GAPDH	M. Musculus	AATGTCAGCAATGCATCCTG	ATGGACTGTGGTCATGAGCC
pri-let-7a/f/d	H. Sapiens	GCGCGAGGAAACCAGGAT	GCACCACAGGAAGGCTTTTTT
LIN28A	H. Sapiens	CTGGAATCCATCCGTGTCA	TCTAGACCTCCACAGTTGTAGCA
LIN28B	H. Sapiens	GCCCCTTGGATATTCCAGTC	AATGTGAATTCCACTGGTTCTCCT
ITS1	H. Sapiens	GTTCGCTCGCTCGTTCGT	CACGCCCTTCTTTCTCTTTC

Supplementary figure legends

Supplementary Fig. S1. *let-7* miRNA levels in RPMI-8226 cells after *DIS3* knockdown using an additional *DIS3* shRNA (sh2). (A) Western blot analysis assaying DIS3 expression in RPMI infected with a scrambled shRNA (ctrl) or *DIS3* shRNA (sh2). Lamin B was used as loading control. (B) Expression of the *let-7* miRNA members (*a, b, f, g*) was assayed by qRT-PCR 72 hours after infection with scrambled (ctrl) or *DIS3*-specific shRNA (sh2). Results are normalized over *RNU6B*. Bars represent SDs (n=2 indipendent experiments). *P< 0.05 using two-tailed Student's t test.

Supplementary Fig. S2. MYC and RAS proteins levels after *DIS3* knockdown in U2OS cells. (A) Representative blot of DIS3, MYC and RAS proteins in U2OS cells, 72 hours after infection with scrambled (ctrl) or *DIS3*-specific shRNA (sh). (B) *MYC* and *RAS* mRNA levels normalized over *GAPDH* in the same cells of panel. Bars represent SDs (n=2 indipendent experiments).

Supplementary Fig. S3. MYC and RAS proteins levels in RPMI cells after *DIS3* knockdown using an additional *DIS3* shRNA. (A) Representative blot for DIS3, MYC and RAS proteins 72 hours after infection with a second shRNA for *DIS3* (sh2). (B) *MYC* and *RAS* mRNA levels normalized over *GAPDH* in the same cells of panel A. Bars represent SDs (n=2 indipendent experiments).

Supplementary Fig. S4. MYC and RAS proteins levels after *DIS3* knockdown in HEK-293T cells. (A) Representative blot for DIS3, MYC and RAS proteins in HEK-293T cells, 72 hours after infection with scrambled (ctrl) or *DIS3*-specific shRNA (sh). (B) *MYC* and *RAS* mRNA levels normalized over *GAPDH* in the same cells of panel A Bars represent SDs (n=2 indipendent experiments).

Supplementary Fig. S5. *DIS3* silencing induces transformation. Western blot of DIS3 levels (A) and focus formation assay (B) of NIH3T3 cells infected with scrambled shRNA (ctrl) or with an additional murine *DIS3* shRNA (sh2). Colonies were counted from 3 independent platings. The error bars represent SD **P < 0.005 using two-tailed Student's t test.

Supplementary Fig. S6. LIN28B levels and response to *DIS3* knockdown in different cell lines. Western blot of DIS3 and LIN28B protein levels in KMS12 and RPMI MM cells, U2OS and HEK-293T cells, 72 hours after infections. Protein extracts equivalent to 200,000 cells (A) and to 1,000,000 cells (B) were loaded in each lane. Lamin B was used as loading control.

Supplementary Fig. S7. *DIS3* silencing increases *LIN28B* mRNA in the NIH3T3 mouse fibroblast cell line. *LIN28B* mRNA levels were assessed by qRT-PCR with respect to *GAPDH* expression, 72 hours after infection. Bars represent SDs (n=2 indipendent experiments). *P< 0.05 using two-tailed Student's t test.

Supplementary Fig. S8. *DIS3* silencing increases LIN28B levels. (A) *LIN28A* and *LIN28B* mRNA levels assessed by qRT-PCR with respect to *GAPDH* expression, 72 hours after infection with the additional *DIS3* shRNA (sh2) in KMS12. Bars represent SDs (n=2 indipendent experiments). *P< 0.05 using two-tailed Student's t test. (B) Western blot of DIS3 and LIN28B protein level in KMS12, 72 hours after infections. Lamin B was used as loading control.

Supplementary Fig. S9. *DIS3* silencing in HEK-293T cells does not increase LIN28B levels. (A) *LIN28A* and *LIN28B* mRNA levels assessed by qRT-PCR with respect to *GAPDH* expression, 72 hours after infection with scrambled (ctrl) or *DIS3*-specific shRNAs (sh) in HEK-293T cells. Bars represent SDs (n=2 indipendent experiments). (B) Western blot of DIS3 and LIN28B protein levels in HEK-293T cells, 72 hours after infection. Lamin B was used as loading control.

Supplementary Fig. S10. *DIS3* knockdown efficiency in cells silenced for *LIN28B*. Western blot of DIS3 protein levels in RPMI cells (A) and U2OS cells (B), 72 hours after the second infection. Lamin B was used as loading control.

Supplementary Fig. S11. DIS3 controls *let-7* through LIN28B. *LIN28B* mRNA (left panel) and *let-7-a* and *let-7-g* (right panel) levels in U2OS cells infected with a scrambled shRNA (ctrl) or with a *LIN28B* shRNA followed, after 3 days, by the infection with a scrambled shRNA (ctrl) or with a *DIS3* shRNA4 (DIS3 sh). *LIN28B* and l*et-7* levels were measured 5 days after the second infection and normalized over *GAPDH* and *RNU6B* respectively. Bars represent SDs (n=2 indipendent experiments). *P< 0.05; **P < 0.005 using two-tailed Student's t test.

Supplementary Fig. S12. ARE motifs (AUUUA pentamers) in the human *LIN28B* 3' UTR (AREsite database (http://rna.tbi.univie.ac.at/cgi-bin/AREsite.cgi)). (A). 15 ARE motifs were identified in the 3' UTR of *LIN28B*. (B) Detailed summary of the analysis results for the *LIN28B* transcript. (C) Figure highlighting detected ARE motif in the 3e 3igure *LIN28B* transcript. (D) Overview of the conservation analysis. Black circles (genomic alignments) and boxes (transcript alignments) indicate ARE motifs found in the sequence of the corresponding species.

Supplementary Fig. S13. Knockdown of SKI and TRAMP complexes helicases. U2OS were transfected with a pool of scrambled siRNAs (ctrl) or a pool of siRNAs targeting the *SKI2* helicase of cytoplasmic exosome component SKI (SKI) or the *MTR4* helicase of nuclear exosome component TRAMP (TRAMP)**.** Cells were collected 48 hours after transfection. Lamin B was used as loading control.

Supplementary Methods

Lentiviral vectors and infections

To knockdown *DIS3* expression, short hairpin sequences targeting either the human or murine *DIS3* were cloned into the lentiviral vector pLKO.1 (Open Biosystem, Huntsville, AL, USA) and cells infected. Non-targeting, scrambled shRNA was used as negative control (Sigma, St. Louis, MO,

USA). Sequences are reported in Supplemental Table S1 For viral production lentiviral plasmid DNA was cotransfected with pRSV-REV, pMD2 VSVG and pMDLg-pRRE plasmids with $CaCl_2$ into 293T packaging cells. After 48 hours, culture supernatants containing the released virus were collected and used to transduce cells. The day after cells infected were selected by treating with puromycin for 3 days (3 μg/ml for NIH3T3, 2 μg/ml for RPMI-8226, 4 μg/ml for KMS12-BM, 2μg/ml for U2OS).

Western Blotting

Cells were lysed in Laemmli buffer (SDS 2%, DTT 100mM, Tris-HCl pH 6.8 0.08M, Glycerol 10%, BromoFenol Blue) were subjected to sodium dodecyl sulfate-polyacrylamide gel electrophoresis and transferred to Amersham Hybond ECL nitrocellulose membrane (GE Healthcare Life Science, Little Chalfont, Buckinghamshire, UK). Antibodies and dilutions are described in supplemental table S2. Proteins in blots were quantified using densitometry function of the imageJ software (http://imagej.nih.gov/ij/index.html), normalized to Lamin B within the same sample and expressed as fold change compared to control.

qRT-PCR

Retrotranscription on RNA purified with TRIzol® was performed using SuperScript® III Reverse Transcriptase by Invitrogen. Comparative real-time PCR was performed in triplicate on ViiA™ 7 Real-Time PCR System using the SYBR green PCR Master Mix Protocol. Expression of *MYC* and *RAS* was calculated using the ΔΔCT method whereas the expression of *LIN28A* and *LIN28B* was calculated using the ΔCT method. *GAPDH* was used as internal control.

For individual miRNA abundance assays Trizol isolated RNA was retrotranscribed in a final volume of 15 μl according to manufacturer's protocol (TaqMan® MicroRNA Reverse Transcription Kit, Applied Biosystems) with stem-looped primers specific for individual mature miRNAs *let-7a* (assay ID: 000377), *let-7b* (000378), *let-7f* (000382), *let-7g* (000383) and *RNU6B* (001093). qRT-PCR was performed in triplicate using the manufacturer's kit probe and the TaqMan®Universal PCR Master Mix reagent. miRNA expression was calculated using the ΔΔCT

method and *RNU6B* as internal control. Pri-miRNA levels were assayed according to what describe in (1) Primers sequences are provided in Table S3.

Northern blot

Northern blot for let-7 miRNA was performed as previously reported with some modifications (2). Briefly 10 μg of RNA purified with Trizol and treated with DNase were fractionated on a 15% denaturing polyacrylamide TBE-Urea Gel (Invitrogen Carlsbad, CA, USA). Gel was pre-run at 300 V for 60 minutes in TBE 1x and then samples were run at 180 V for 50 minutes. The RNA was electrotrasferred to Hybond-N+ nylon membrane (GE Healthcare Life Science, Little Chalfont, Buckinghamshire, UK) at 30V for 1 h and cross-linked with freshly prepared EDC reagent at 60°C for 90 minutes as described (3). Membrane was then thoroughly rinsing with distillated water to remove residual cross-linking solution, rolled with RNA side in, insert into hybridization bottle and pre-hybridized for 30 minutes in 15 ml of UltraHyb buffer (Ambion, Naugatuck, USA) at 37°C. *let-7g* and *U6* miRCURY LNA detection probes (Exiqon, Vedbaek, Denmark) were denatured at 95°C for 1 minute, chilled in ice and added to the hybridization buffer to yield a final concentration of 0.5 nM. Membrane was hybridized at 37°C overnight with slow rotation. After that, hybridization buffer was discarded and membrane was washed twice with 30 ml of low stringent buffer (SSC 2x, 0,1% SDS) for 15 minutes at 37°C and twice with 30 ml high stringent buffer (SSC 0,1X, SDS 0,1%) for 5 minutes at 37°C. Membrane was then briefly rinsed with 30 ml of Washing Buffer (Roche Diagnostic, Mannheim, Germany) at 37°C for 10 minutes and incubated in Blocking Buffer (Roche Diagnostic, Mannheim, Germany) at room temperature. After 3 hrs, Blocking Buffer was replaced by a DIG antibody solution prepared by diluting Anti-Digoxigenin-AP, Fab fragments (Roche Diagnostic, Mannheim, Germany) 1:15,000 in Blocking Buffer. Membrane was incubated at room temperature for 30 minutes and washed in DIG Washing Buffer (Roche Diagnostic, Mannheim, Germany) four times for 15 minutes each at room temperature. Subsequently on the surface of membrane incubated in Detection Buffer (Roche Diagnostic, Mannheim, Germany) for 5 minutes at room temperature, removed from bottle and placed on Saran

wrap, 1 ml of CSPD reagent (Roche Diagnostic, Mannheim, Germany) diluted 1:100 in Detection Buffer was applied. After 5 minutes of incubation at room temperature in the dark, extra CSPD buffer was squeeze out, membrane was sealed in the saran wrap, incubated in the dark at 37°C for 15 minutes and exposed to X-ray film for 30 minutes at room temperature.

AU-rich elements analysis

Investigation of AU-rich elements in 3'-UTR of human LIN28B mRNA was performed using a publicly available database (http://rna.tbi.univie.ac.at/AREsite) (4).

Supplementary references

1. Chang TC*, et al.* (2009) Lin-28B transactivation is necessary for Myc-mediated let-7 repression and proliferation. *Proc Natl Acad Sci U S A* 106(9):3384-3389.
2. Kim SW*, et al.* (2010) A sensitive non-radioactive northern blot method to detect small RNAs. *Nucleic Acids Res* 38(7):e98.
3. Pall GS & Hamilton AJ (2008) Improved northern blot method for enhanced detection of small RNA. *Nat Protoc* 3(6):1077-1084.
4. Gruber AR, Fallmann J, Kratochvill F, Kovarik P, & Hofacker IL (2011) AREsite: a database for the comprehensive investigation of AU-rich elements. *Nucleic Acids Res* 39(Database issue):D66-69.
